# CFTR Modulators: The Changing Face of Cystic Fibrosis in the Era of Precision Medicine

**DOI:** 10.3389/fphar.2019.01662

**Published:** 2020-02-21

**Authors:** Miquéias Lopes-Pacheco

**Affiliations:** Biosystems & Integrative Sciences Institute, Faculty of Sciences, University of Lisbon, Lisbon, Portugal

**Keywords:** cystic fibrosis, CFTR mutations, personalized medicine, drug development, high-throughput screening, cell models, clinical trials, lung

## Abstract

Cystic fibrosis (CF) is a lethal inherited disease caused by mutations in the CF transmembrane conductance regulator (*CFTR*) gene, which result in impairment of CFTR mRNA and protein expression, function, stability or a combination of these. Although CF leads to multifaceted clinical manifestations, the respiratory disorder represents the major cause of morbidity and mortality of these patients. The life expectancy of CF patients has substantially lengthened due to early diagnosis and improvements in symptomatic therapeutic regimens. Quality of life remains nevertheless limited, as these individuals are subjected to considerable clinical, psychosocial and economic burdens. Since the discovery of the *CFTR* gene in 1989, tremendous efforts have been made to develop therapies acting more upstream on the pathogenesis cascade, thereby overcoming the underlying dysfunctions caused by CFTR mutations. In this line, the advances in cell-based high-throughput screenings have been facilitating the fast-tracking of CFTR modulators. These modulator drugs have the ability to enhance or even restore the functional expression of specific CF-causing mutations, and they have been classified into five main groups depending on their effects on CFTR mutations: potentiators, correctors, stabilizers, read-through agents, and amplifiers. To date, four CFTR modulators have reached the market, and these pharmaceutical therapies are transforming patients' lives with short- and long-term improvements in clinical outcomes. Such breakthroughs have paved the way for the development of novel CFTR modulators, which are currently under experimental and clinical investigations. Furthermore, recent insights into the CFTR structure will be useful for the rational design of next-generation modulator drugs. This review aims to provide a summary of recent developments in CFTR-directed therapeutics. Barriers and future directions are also discussed in order to optimize treatment adherence, identify feasible and sustainable solutions for equitable access to these therapies, and continue to expand the pipeline of novel modulators that may result in effective precision medicine for all individuals with CF.

***In Memoriam:*** The author dedicates this review article to his youngest brother, Nilo Lopes Pacheco, who fought against CF for 26.5 years.

## Introduction

Mutations in the cystic fibrosis transmembrane conductance regulator (*CFTR*) gene cause cystic fibrosis (CF)—the most common life-threatening autosomal recessive disease in Caucasian populations (Lopes-Pacheco, 2016). *CFTR* encodes a cAMP-dependent, phosphorylation-activated anion channel that transports chloride and bicarbonate across the apical plasma membrane (PM) of epithelial cells (Riordan, 2005; Saint-Criq and Gray, 2017). Furthermore, CFTR modulates the activity of other ion channels, such as the epithelial sodium channel (ENaC) (Saint-Criq and Gray, 2017; Moore and Tarran, 2018). The absence or dysfunction of the CFTR protein at the PM leads to an impaired transepithelial balance of ions and fluid in cells of the sweat glands, airways, intestine, and pancreas, among other organs. Although CF is a multi-organ disease, the respiratory disorder represents the major cause of morbidity and mortality of these patients. A vicious cycle of mucus buildup in the airways, chronic inflammation, and recurrent infections leads to epithelial damage, tissue remodeling and progressive deterioration of lung function, ultimately resulting in respiratory failure (refer to Figure 4 in Lopes-Pacheco, 2016).

Archeological estimates indicate that the most prevalent CF-causing mutation, the deletion of a phenylalanine at position 508 (F508del), originated in Western Europe during the Early Bronze Age (Farrell et al., 2018). Although there are some archaic references regarding “children whose brow had salty taste when kissed and prematurely died”, CF remained uncharacterized until the 1930s. The first pathological description of the disease came in 1938 when Dorothy Anderson recognized CF as a separate entity from celiac syndrome after autopsy studies of malnourished infants, being then known as “cystic fibrosis of the pancreas” (Anderson, 1938). Another critical discovery was reported by Paul di Sant' Agnese in 1953 when he noticed that CF patients demonstrated an abnormal excess of salt in the sweat during a heat wave in New York (Di Sant'Agnese et al., 1953). Nevertheless, the decreased chloride transport and increased sodium reabsorption was described as a basic defect in CF epithelia in the 1980s by experiments using sweat duct cells (Knowles et al., 1983; Quinton, 1983; Boucher et al., 1986). Such findings served as basis for the sweat chloride test extensively used nowadays in CF diagnosis. Finally, the correlation between CF and the *CFTR* gene was discovered in 1989 when the gene was cloned by using chromosome walking and jumping, and linkage disequilibrium analysis (Kerem et al., 1989; Riordan et al., 1989; Rommens et al., 1989). Soon after, some reports demonstrated that CFTR-dependent chloride secretion could be restored by transfecting cells derived from CF patients with wild type (WT)-CFTR cDNA (Drumm et al., 1990; Rich et al., 1990).

The extensive knowledge obtained over this research path has enabled the early diagnosis and the discovery of more efficient and sophisticated therapies, resulting in increased life expectancy. In fact, the mean age of survival of CF has risen from early childhood in the 1960s to 40–50 years currently in several countries, although it still is much lower in certain regions worldwide. The “backbone” of CF treatment is symptomatic, focusing on the compensation of pancreatic insufficiency and intestinal malabsorption with pancreatic enzymes, fat-soluble vitamins, and high-calorie ingestion, as well as slowing lung function deterioration with physical and inhaled therapies to enhance airway clearance, anti-inflammatory drugs, and antibiotic therapy to eradicate infections (Cohen-Cymberknoh et al., 2011; Athanazio et al., 2017; Castellani et al., 2018). At late stages of disease, lung transplantation remains the only feasible intervention (Stephenson et al., 2017; Ramos et al., 2019), although still presenting a risk of cellular rejection (Calabrese et al., 2015). Furthermore, several comorbidities that were rare or not previously observed, including CF-related diabetes, metabolic bone and kidney disorders, and certain types of cancer, have become increasingly common as CF patients' lives have lengthened (Ronan et al., 2017). A tremendous effort has been made to continue optimizing the therapeutic regimens and the multidisciplinary healthcare in order to further enhance CF patients' life expectancy. Quality of life has also improved but patients are still subjected to substantial clinical, psychosocial and economic burdens.

Novel therapeutic approaches acting more upstream on the pathogenesis cascade have emerged (Lopes-Pacheco et al., 2019), including precision medicine with the discovery of drugs (termed CFTR modulators) that rectify the underlying defects of certain CFTR mutations. To date, four CFTR modulators have reached the market for the treatment of patients carrying specific CF-causing mutations and these breakthroughs have paved the way for the development of novel pharmacotherapies, which are currently under experimental and clinical investigations. This review provides a summary of recent developments in CFTR-directed therapeutics and sheds light on barriers that must be overcome for precision medicine efficiently to reach all individuals with CF.

## A Brief Overview of CFTR Biology

### *CFTR* Gene and mRNA

**Figure 1:**
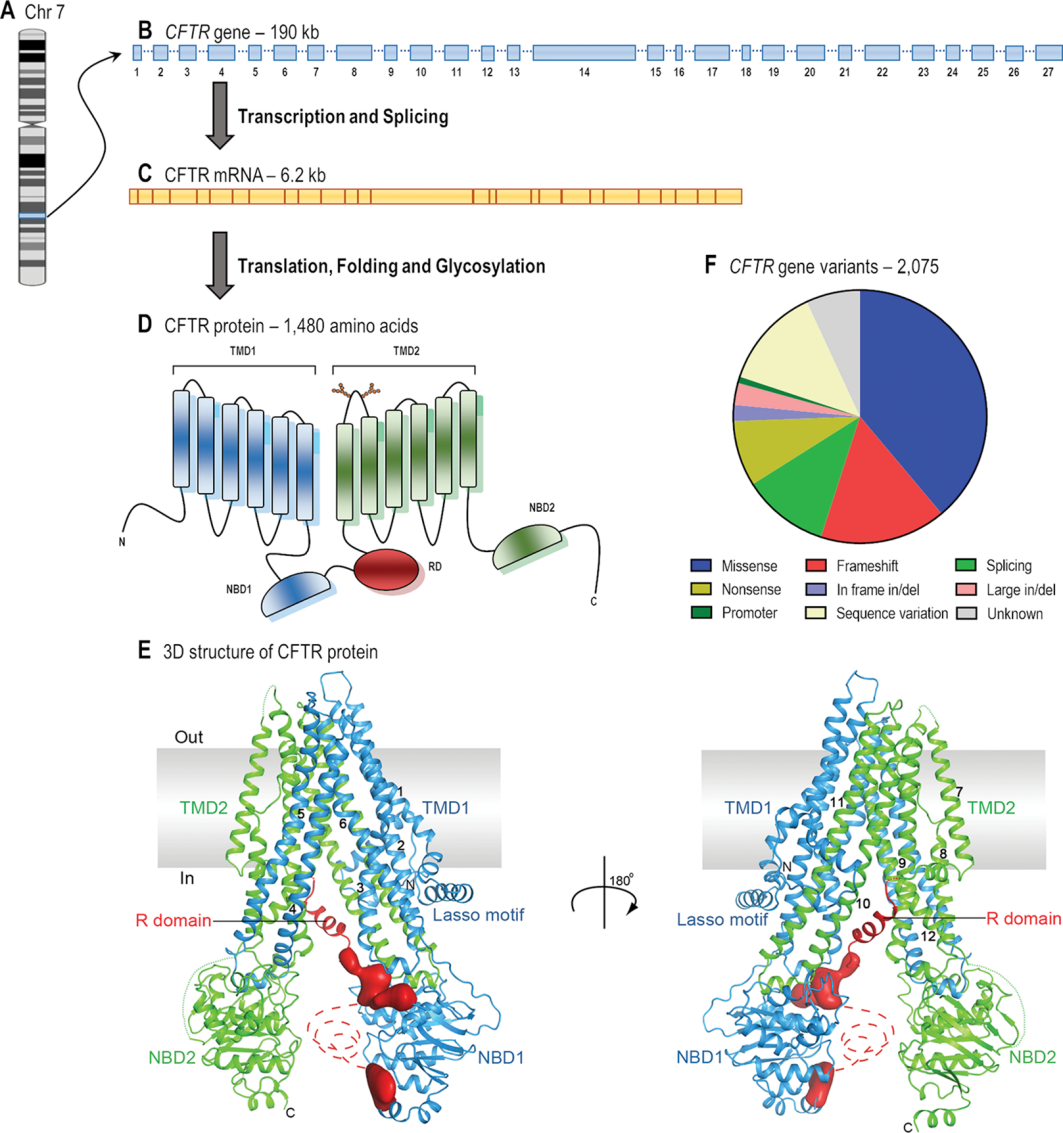
From gene to protein structure. **(A)** CF transmembrane conductance regulator (*CFTR*) gene is located on the long arm of chromosome (Chr) 7. **(B)** The gene contains 27 exons and spans approximately 190 kb of human genomic DNA. **(C)** The mRNA is 6.2 kb long including the untranslated regions (adapted from Collins, 1992). **(D)** The protein forms a chloride/bicarbonate channel composed of five domains: two transmembrane domains (TMD1 and TMD2), two nucleotide-binding domains (NBD1 and NBD2) and a regulatory domain (RD) (adapted from Lopes-Pacheco, 2016). **(E)** The overall structure of human CFTR in the dephosphorylated, ATP-free conformation (adapted from Liu et al., 2017 with permission from Prof. J. Chen). **(F)** The 2,075 *CFTR* gene variants that have so far been reported consist of missense (38.9%), frameshift (16.1%), splicing (11.1%), and nonsense (8.4%) mutations; in-frame (2.1%) and large (2.8%) deletions or insertions; promoter mutations (0.9%); and possibly non-pathogenic variants (13.0%) (adapted from CFTR1 Database).

*CFTR* is a long gene located on the long arm of chromosome 7, specifically in 7q31.2 (**Figure 1**). It is composed of 27 coding exons, spanning approximately 190 kb of human genomic DNA that is transcribed into a CFTR mRNA of 6.2 kb (Collins, 1992). Over 2,000 *CFTR* gene variants have been reported in the Cystic Fibrosis Mutation Database (CFTR1 Database). Many of them might be a variation in the DNA sequence eliciting neither a defect in CFTR mRNA or protein nor clinical symptoms. To date, 432 of these variants have been annotated in the Clinical and Functional Translation of CFTR (CFTR2 Database), of which 352 variants have confirmed disease liability and 46 variants have demonstrated variable clinical consequences. Many other variants are still uncharacterized, and *in silico* tools may be useful to predict the cellular and molecular consequences caused by these alterations (Michels et al., 2019; Pereira et al., 2019), while they are not confirmed in cell models. Some variants may also be pathogenic when two or more mutations are in *cis*, i.e., complex allele, thus contributing to the variable clinical phenotypes (Lucarelli et al., 2010; Diana et al., 2016; Pereira et al., 2019) and responsiveness to CFTR modulator therapies.

### CFTR Protein

The CFTR mRNA translates into a 1,480-amino acid protein. Soon after co- and post-translational folding, and core glycosylation in the endoplasmic reticulum (ER), CFTR protein traffics to the Golgi complex, where it is fully glycosylated. Thereafter, it is exported to the apical PM of epithelial cells, where it functions as a chloride and bicarbonate channel (Kim and Skach, 2012; Lukacs and Verkman, 2012).

The CFTR protein has five distinct domains: two transmembrane domains (TMD1 and TMD2), two nucleotide-binding domains (NBD1 and NBD2), and a regulatory domain (RD). Each TMD contains six segments that completely cross the phospholipid bilayer and together they form the channel pore through which anions may flow (Riordan, 2005). The TM segments are joined by three extracellular loops and two intracellular loops in each TMD, and they have critical roles in CFTR biogenesis and in the post-translational folding in order to establish interdomain interactions required to achieve conformational stability (Kim and Skach, 2012). Furthermore, the fourth extracellular loop possesses two N-linked glycosylation sites (N894 and N900), which are assessed by the ER quality control during the protein folding process. These glycans are modified upon traversing the Golgi complex and may interact with extracellular macromolecules when the protein is located at the PM (Glozman et al., 2009; Lukacs and Verkman, 2012). The NBDs and the RD are exposed to the cytosol and are rich in charged residues. The NBDs present highly conserved sequence for ATP binding and hydrolysis, while the RD is highly disordered and has multiple consensus sequences containing serines and threonines for phosphorylation by protein kinase A (PKA) and protein kinase C (PKC). ATP and cAMP-dependent PKA and PKC phosphorylation induce alterations in CFTR protein conformation, thus allowing anion conductance through the pore (Collins, 1992; Chappe et al., 2003; Saint-Criq and Gray, 2017). Among the 48 members of the human ATP-binding cassette (ABC) transporter family, CFTR (also known as ABCC7) is the unique one that possesses a RD and functions as an anion channel. Novel insights into channel opening and closure mechanisms have recently been elucidated with electron cryomicroscopy of the phosphorylated and dephosphorylated protein state (Liu et al., 2017; Zhang et al., 2017; Fay et al., 2018; Zhang et al., 2018; Liu F. et al., 2019). While these structures have not played a role in the development of CFTR modulators to date, such findings will be greatly useful for the identification of hotspots for drug-binding and for the application of rational design of next-generation modulator drugs based on the CFTR structure.

## CF-Causing Mutations and Progress in Precision Medicine

**Figure 2:**
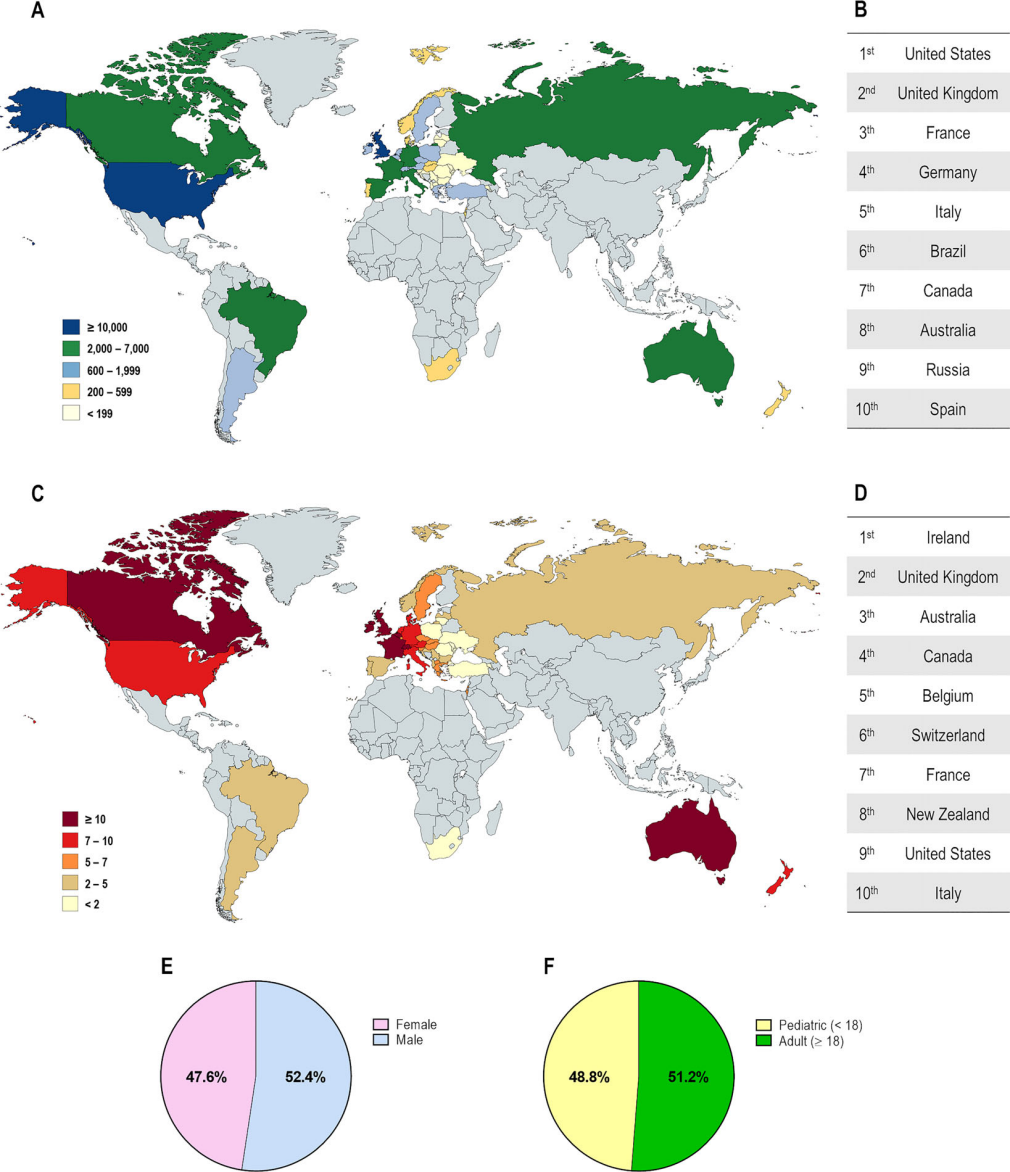
Demography of cystic fibrosis patients in different countries. **(A)** Distribution according to the total number of patients registered. **(B)** Top 10 countries with the highest number of patients registered. **(C)** Distribution according to the estimated prevalence of patients per 100,000 habitants. **(D)** Top 10 countries with the highest number of patients per 100,000 habitants. Global distribution by gender **(E)** and by age **(F)**. [Data compiled from the last Patient Registry Report in Argentina (Pereyro et al., 2018), Australia (Cystic Fibrosis Australia, 2018), Brazil (Brazilian Cystic Fibrosis Study Group, 2019), Canada (Cystic Fibrosis Canada, 2019), Europe (European Cystic Fibrosis Society, 2019), New Zealand (Cystic Fibrosis New Zealand, 2019), South Africa (Zampoli et al., 2019), UK (Cystic Fibrosis Trust, 2019), and the USA (Cystic Fibrosis Foundation, 2019)].

**Figure 3:**
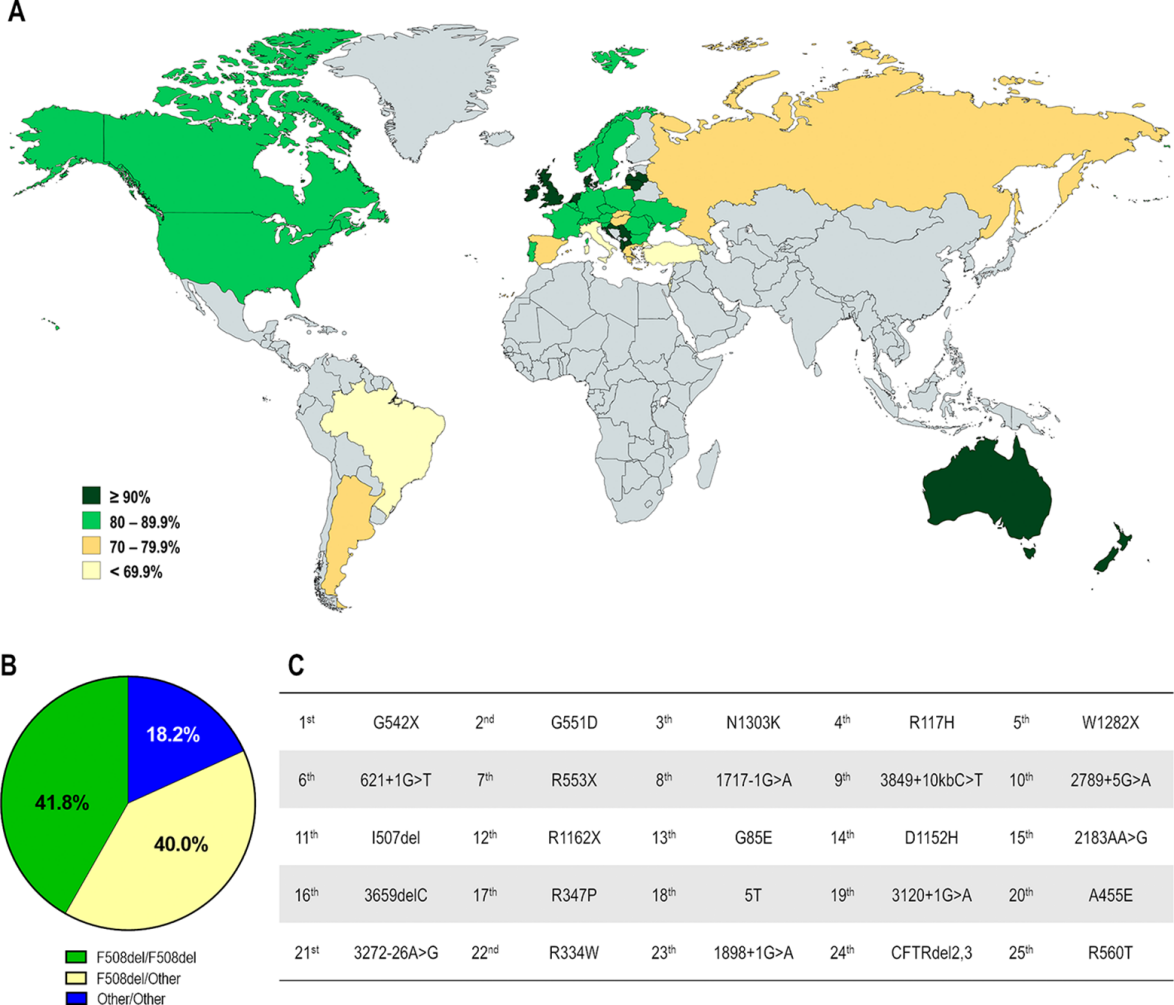
Demography of CF transmembrane conductance regulator (CFTR) mutations in different countries. **(A)** Distribution according to the percentage of patients carrying the F508del mutation in at least one allele. **(B)** Global distribution by CF genotype: F508del-homozygous, F508del-heterozygous and carrying non-F508del mutations in both alleles. **(C)** Top 25 most prevalent non-F508del CFTR mutations considering the whole CF population. [Data compiled from the last Patient Registry Report in Argentina (Pereyro et al., 2018), Australia (Cystic Fibrosis Australia, 2018), Brazil (Brazilian Cystic Fibrosis Study Group, 2019), Canada (Cystic Fibrosis Canada, 2019), Europe (European Cystic Fibrosis Society, 2019), New Zealand (Cystic Fibrosis New Zealand, 2019), UK (Cystic Fibrosis Trust, 2019), and the USA (Cystic Fibrosis Foundation, 2019), and CFTR2 Database].

CF affects over 90,000 individuals and they are heterogeneously distributed worldwide (**Figure 2**). The F508del is the most prevalent CF-causing mutation, affecting approximately 82% of the CF population (**Figures 3A, B**). This mutation leads to CFTR protein misfolding that is arrested by the ER quality control, thus precluding its processing and trafficking to the PM, being instead targeted and prematurely degraded by proteasomes (Cheng et al., 1990; Jensen et al., 1995). Nevertheless, a small fraction of the mutant protein may evade the quality control checkpoints and reach the PM; however, it still presents a defective gating (Dalemans et al., 1991; Haws et al., 1996) and a considerable reduction in protein stability (Sharma et al., 2004; Swiatecka-Urban et al., 2005; Okiyoneda et al., 2010).

The F508del mutation accounts for approximately 70% of CF alleles and other CFTR mutations are responsible for the remaining ones. Notably, most are rare or demonstrate a certain frequency in specific regions, and only six mutations present a prevalence ≥1% while approximately 50 mutations have a prevalence ≥0.1% considering the whole CF population (**Figure 3C**).

**Figure 4:**
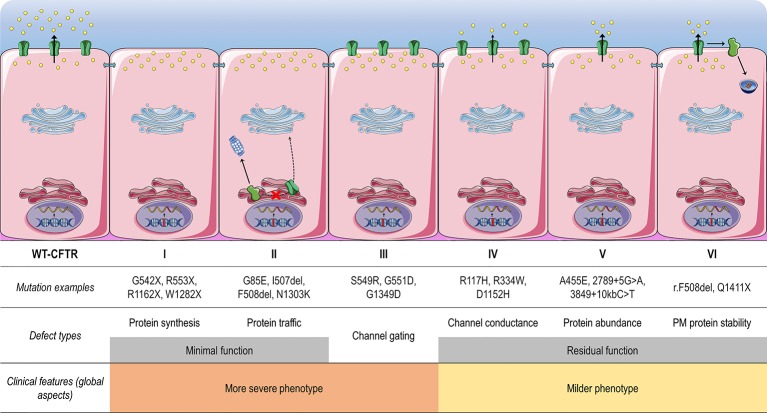
Classes of CF transmembrane conductance regulator (CFTR) mutations. *Class I* mutations lead to no protein synthesis or translation of shortened, truncated forms. They result from splice site abnormalities, frameshifts due to deletions or insertions, or nonsense mutations, which generate premature termination codons (PTCs). *Class II* mutations lead to a misfolding protein that fails to achieve conformational stability in the endoplasmic reticulum and then does not traffic to the plasma membrane (PM), being instead prematurely degraded by proteasomes. *Class III* mutations lead to a gating channel defect due to impaired response to agonists, although the protein is present at the PM. *Class IV* mutations lead to a channel conductance defect with a significant reduction in CFTR-dependent chloride transport. *Class V* mutations lead to a reduction in protein abundance of functional CFTR due to reduced synthesis or inefficient protein maturation. They result from alternative splicing, promoter or missense mutations. *Class VI* mutations lead to reduced protein stability at the PM, which results in increased endocytosis and degradation by lysosomes, and reduced recycling to the PM. Mutations in *classes I* and *II* are also known as minimal function mutations since they demonstrate no to very little CFTR function, while those in *classes IV, V*, and *VI* are known as residual function mutations since they demonstrate some CFTR function, although it is lower compared to the wild type (WT)-CFTR. (adapted from Lopes-Pacheco, 2016).

**Figure 5:**
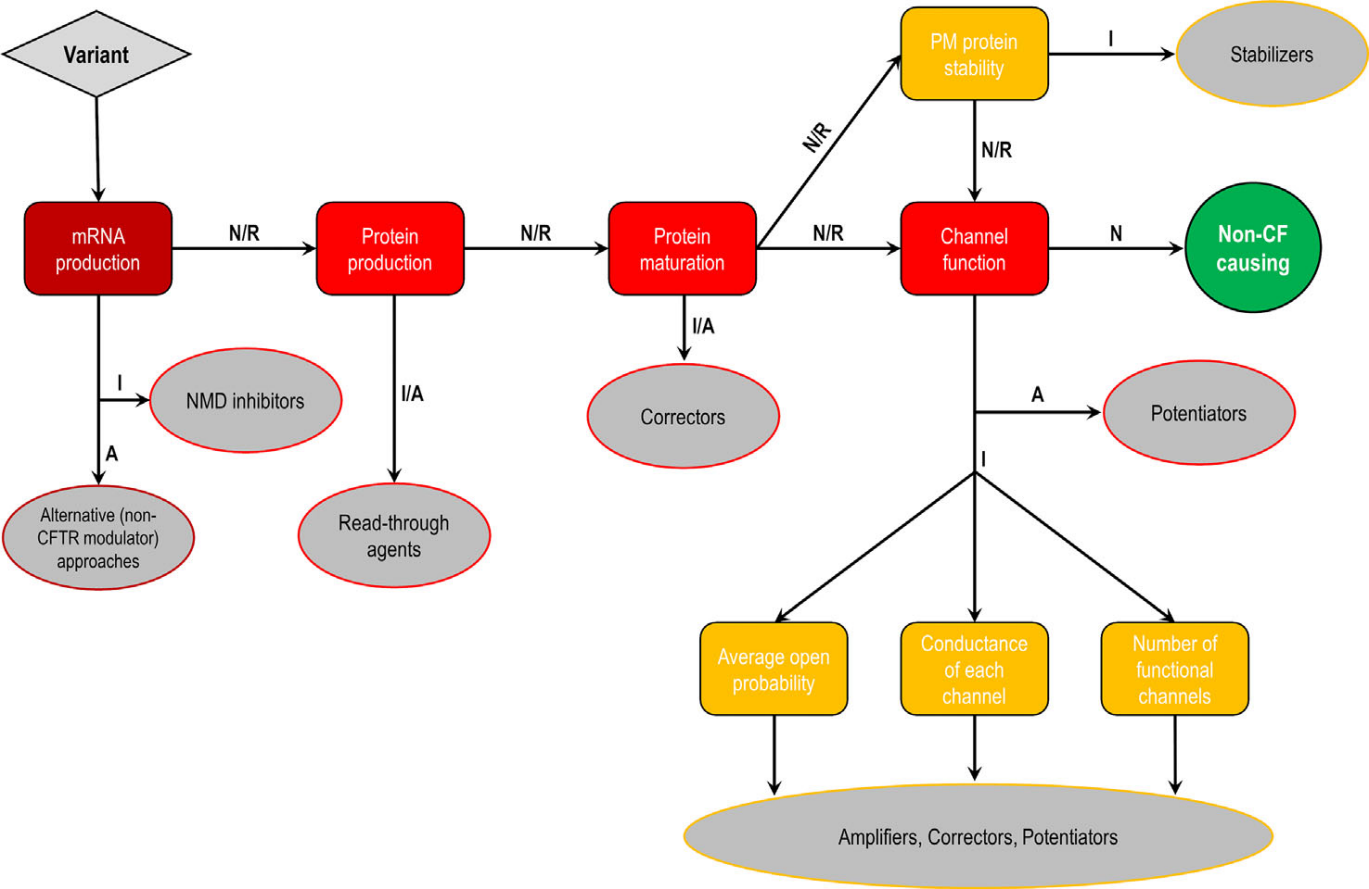
Cellular and molecular defects and potential CF transmembrane conductance regulator (CFTR) modulator approaches. Flowchart demonstrating the steps to identify each cellular and molecular defect of a *CFTR* gene variant and potential therapeutic approaches to correct each of these defects. Abbreviations: A, abrogated; I, impaired; N, normal; R, rescued.

CFTR mutations may impair mRNA and protein expression, function, stability or a combination of these, and as such they have been stratified into different classes according to the primary molecular defect. The classification has historically been evolving according to the gained knowledge (Collins, 1992; Welsh and Smith, 1993; Wilschanski et al., 1995; Haardt et al., 1999; Rowe et al., 2005), and the current scheme is composed of six classes (**Figure 4**), although a seventh class has been proposed to separately consider large deletions that may abrogate production of CFTR mRNA (De Boeck and Amaral, 2016; Marson et al., 2016). Another system has also been proposed to take into account the pleiotropic defects of many CFTR mutations, including the F508del (Veit et al., 2016a). Although limitations in the classification system are evident, it has been useful in understanding the distinct cellular and molecular defects of different CFTR mutations as well as in the development of pharmacotherapies for specific defects (**Figure 5**).

Mutations in classes I, II, and III are usually associated with a classical and more severe disease, while those in classes IV, V, and VI are related to milder (or atypical) phenotypes. Individuals with CF may nevertheless carry different CFTR mutations on the two alleles, leading to thousands of possible combinations of CF genotypes. Noteworthy, CFTR mutations may differently respond to the same intervention (e.g., correction by low temperature or by chemical compounds), even for those classified as belonging to the same defect class (Rapino et al., 2015; Dekkers et al., 2016a; Dekkers et al., 2016b; Lopes-Pacheco et al., 2016; Lopes-Pacheco et al., 2017; Han et al., 2018; Awatade et al., 2019). Therapeutic responses may also differ between individuals carrying the same CF genotypes (Boyle et al., 2014; Donaldson et al., 2018a; Keating et al., 2018; Matthes et al., 2018). In fact, several other factors exert influence on disease severity beyond CFTR mutations, such as gene modifiers and epigenetic factors, social and economic status, patient's lifestyle, and adherence to therapies (Oates and Schechter, 2016; O'Neal and Knowles, 2018). Primary, reprogrammed and engineered human cell models have become important tools to identify novel pharmacotherapies. The effects of certain therapies may also be exploited at an individual level in *ex vivo* patient-derived specimens, such as primary bronchial/nasal epithelial cells, and intestinal/respiratory organoids (Fulcher and Randell, 2013; Dekkers et al., 2016a; Dekkers et al., 2016b; Pranke et al., 2017; Awatade et al., 2018; Brewington et al., 2018; Chen et al., 2018; Berkers et al., 2019; Merket et al., 2019). As these cell models recapitulate several features of the parental organ, they are useful to understand the impact of genetic factors on individual disease and predict clinical efficacy of therapies.

**Figure 6:**
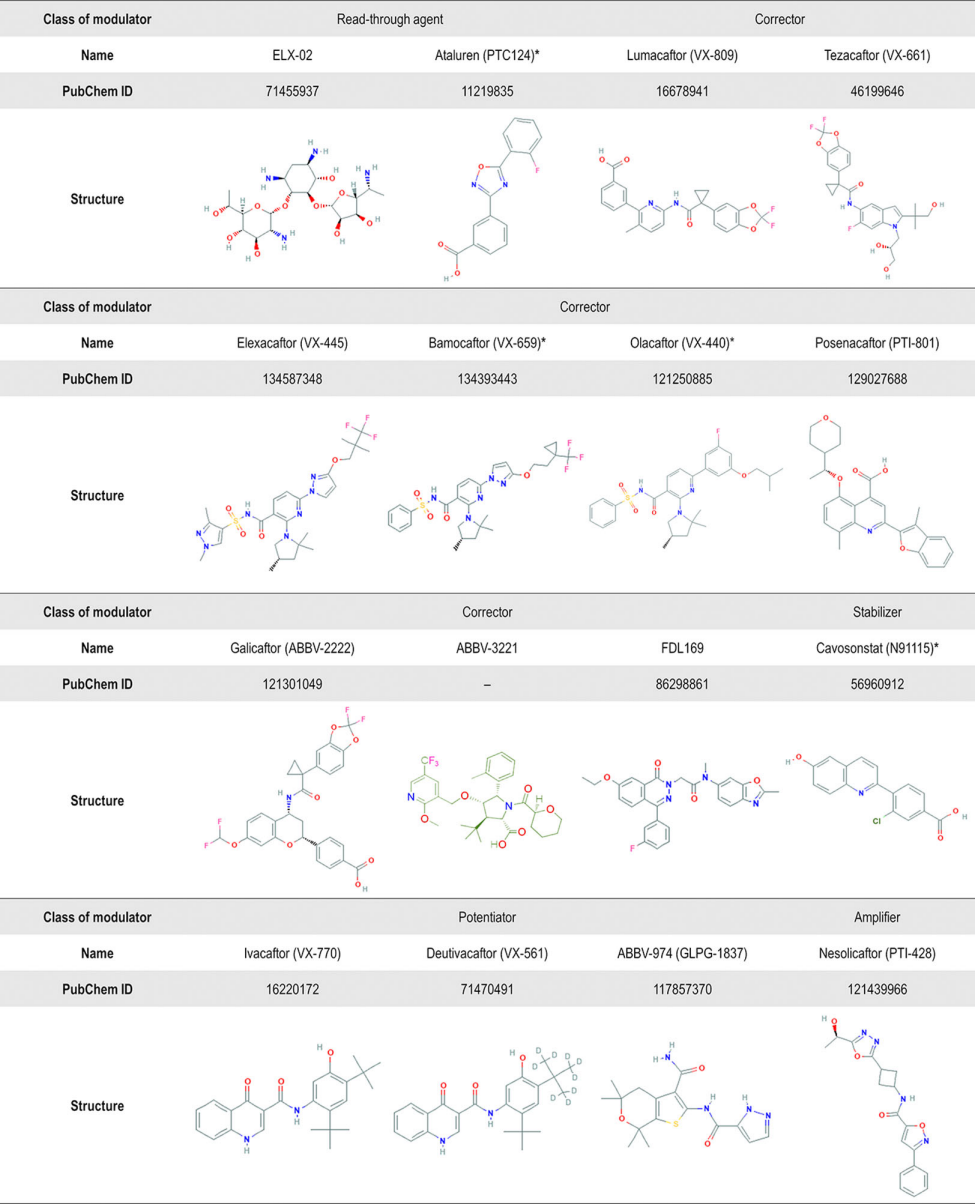
Chemical structure of several CF transmembrane conductance regulator (CFTR) modulators tested in clinical trials or currently in the market. Advances in high-throughput screening technologies have been enabling the identification of small-molecules from different chemical series. In addition to the compounds displayed in this figure, the compounds VX-121, ABBV-2737, ABBV-3067, FDL176, and PTI-808 are also under investigation in clinical trials, but the chemical structures are still not available on the PubChem or DrugBank. *Clinical development has been discontinued.

Numerous libraries of compounds have been screened by distinct high-throughput screening (HTS) methods and using several cell models. These experimental approaches have been contributing to the identification of small-molecules from different chemical series (Pedemonte et al., 2005a; Van Goor et al., 2009; Van Goor et al., 2011; Phuan et al., 2014; Phuan et al., 2015; Liang et al., 2017; Giuliano et al., 2018; Van der Plas et al., 2018; Veit et al., 2018; Wang et al., 2018; Berg et al., 2019; De Wilde et al., 2019; Merket et al., 2019) (**Figure 6**). Notably, as cell background may significantly influence the pharmacological rescue of CFTR mutants (Pedemonte et al., 2010), most promising hits should be further validated in well-differentiated cells in order to identify non-cytotoxic and effective compounds that might result in optimal therapeutic benefits in the clinical scenario.

CFTR modulator drugs enhance or even restore the expression, function, and stability of a defective CFTR by distinct manners, and they have been classified into five main groups depending on their effects on CFTR mutations: potentiators, correctors, stabilizers, read-through agents, and amplifiers (Lopes-Pacheco, 2016). To date, four CFTR-directed modulators have reached the market for the treatment of CF patients carrying specific CFTR mutations (Ramsey et al., 2011; De Boeck et al., 2014; Wainwright et al., 2015; Rowe et al., 2017a; Taylor-Cousar et al., 2017; Heijerman et al., 2019; Middleton et al., 2019). Several clinical trials have been completed (**Table 1**) and many others are ongoing (**Table 2**) in extension/observational studies or to evaluate the safety and efficacy of novel modulators. As the experimental and clinical research in the CF field is moving at an accelerated pace, the most recent advances in precision medicine have been summarized below in order to update information previously published in the 2016 Review in *Frontiers in Pharmacology* (Lopes-Pacheco, 2016).

**Table 1:** Publications evaluating safety and efficacy and post-approval observational studies of CF transmembrane conductance regulator (CFTR) modulators in CF patients (*).

ClinicalTrials.gov ID	Phase	Subjects	Age (years)	Drug(s)	Follow up	Reference(s)
NCT00909532	III	G551D in at least one allele	≥12	Ivacaftor	48 weeks	Ramsey et al., 2011; Quittner et al., 2015; Solem et al., 2016; Flume et al., 2018
NCT01225211	II	F508del-homozygous and -heterozygous	≥18	Lumacaftor/Ivacaftor	56 days	Boyle et al., 2014; Rowe et al., 2017b
NCT01531673	II	F508del-homozygous and -heterozygous	≥12	Tezacaftor/Ivacaftor	56 days	Donaldson et al., 2018a
NCT01705145	III	A gating mutation in at least one allele	2–5	Ivacaftor	24 weeks	Davies et al., 2016
NCT01784419	N-of-1	A gating mutation in at least one allele	≥8	Ivacaftor	Cycles of 14 days	McGarry et al., 2017
NCT01807923 and NCT01807949	III	F508del-homozygous	≥12	Lumacaftor/Ivacaftor	24 weeks	Wainwright et al., 2015; Elborn et al., 2016; Flume et al., 2019; McColley et al., 2019
NCT01897233	III	F508del-homozygous	6–11	Lumacaftor/Ivacaftor	24 weeks	Milla et al., 2017
NCT01931839	III	F508del-homozygous	≥12	Lumacaftor/Ivacaftor	96 weeks	Konstan et al., 2017
NCT01937325	IV	G551D in at least one allele	≥16	Ivacaftor	3 months	Edgeworth et al., 2017
NCT01946412	III	A gating mutation in at least one allele	2–5	Ivacaftor	84 weeks	Rosenfeld et al., 2019
NCT02141464	IV	A gating mutation in at least one allele	≥6	Ivacaftor	3 months	Stallings et al., 2018
NCT02275936	I	F508del-homozygous	≥18	Cavosonstat	28 days	Donaldson et al., 2017
NCT02347657	III	F508del-homozygous	≥12	Tezacaftor/Ivacaftor	24 weeks	Taylor-Cousar et al., 2017
NCT02390219	III	F508del-homozygous	≥12	Lumacaftor/Ivacaftor	24 weeks	Taylor-Cousar et al., 2018
NCT02392234	III	F508del-heterozygous with a residual function mutation in *trans*	≥12	Tezacaftor/Ivacaftor	8 weeks	Rowe et al., 2017a
NCT02514473	III	F508del-homozygous	6–11	Lumacaftor/Ivacaftor	24 weeks	Ratjen et al., 2017
NCT02707562	II	G551D in at least one allele	≥18	ABBV-974	4 weeks	Davies et al., 2019
NCT02725567	III	A gating mutation in at least one allele	1–2	Ivacaftor	24 weeks	Rosenfeld et al., 2018
NCT02797132	III	F508del-homozygous	2–5	Lumacaftor/Ivacaftor	24 weeks	McNamara et al., 2019
NCT02807415	IV	F508del-homozygous	≥6	Lumacaftor/Ivacaftor	16 weeks	Graeber et al., 2018
NCT02953314	III	F508del-homozygous and -heterozygous	6–11	Tezacaftor/Ivacaftor	24 weeks	Walker et al., 2019
NCT02965326	IV	F508del-homozygous	≥12	Lumacaftor/Ivacaftor	6 months	Pranke et al., 2019
NCT03029455 and NCT03224351	II	F508del-homozygous and -heterozygous with a residual function mutation in *trans*	≥18	VX-659/Tezacaftor/Ivacaftor	4 weeks	Davies et al., 2018
NCT03045523	II	F508del-heterozygous with a gating mutation in *trans*	≥18	ABBV-2222 on top of Ivacaftor	29 days	Bell et al., 2019
NCT03119649	II	F508del-homozygous	≥18	ABBV-2222	29 days	Bell et al., 2019
NCT03227471	I/II	F508del-homozygous and -heterozygous with a residual function mutation in *trans*	≥18	Elexacaftor/Tezacaftor/Ivacaftor	4 weeks	Keating et al., 2018
NCT03525444	III	F508del-heterozygous with a minimal function mutation in *trans*	≥12	Elexacaftor/Tezacaftor/Ivacaftor	24 weeks	Middleton et al., 2019
NCT03525548	III	F508del-homozygous	≥12	Elexacaftor/Tezacaftor/Ivacaftor	4 weeks	Heijerman et al., 2019
NCT03474042	IIa	F508del-homozygous	≥18	ABBV-2737 on top of lumacaftor/ivacaftor	28 days	van Koningsbruggen-Rietschel et al., 2019

(*)This table is a continuation of Table 3 published in Lopes-Pacheco, 2016.

**Table 2:** Pipeline of CF transmembrane conductance regulator (CFTR) modulators in clinical trials and in the market (^#^).

Phase	Read-through agents	Correctors	Potentiators	Stabilizers	Amplifiers
**I**		ABBV-3221	FDL176		
**II**	ELX-02 (NB124)	ABBV-2222, ABBV-2737, FDL169, VX-121, [Riociguat], [VX-152], [VX-440]	VX-561, ABBV-974, ABBV-3067, [QBW251]	[Cavosonstat (N91115)]	
**III**	[Ataluren (PTC124)]	PTI-801, [VX-659]	PTI-808		PTI-428
**IV/Market**		Lumacaftor (VX-809), Tezacaftor (VX-661), Elexacaftor (VX-445)	Ivacaftor (VX-770)		

[name] Clinical development was discontinued. (^#^) adapted from CFF Drug Development Pipeline.

### Potentiators: Restoring the Channel Gating and Conductance

Around 5% of CF-causing mutations lead to impaired CFTR channel gating or conductance as primary defects (Classes III and IV, **Figure 4**). The R117H, R334W, R347P, and G551D are among the most common mutations that cause such abnormalities and they are found in 1.3%, 0.3%, 0.4%, and 2.1% of CF alleles, respectively (CFTR2 Database). Potentiators are compounds that restore or even enhance the channel open probability, thus allowing for CFTR-dependent anion conductance (Lopes-Pacheco, 2016).

Initial studies demonstrated an enhancement of the open probability of certain CFTR variants in cells by using either chemically modified analogs of ATP or compounds that increase intracellular cAMP levels (Drumm et al., 1991; Illek and Fischer, 1998; Zhuo et al., 2005). However, their use in the clinics is limited due to potential modulation of multiple signaling pathways and physiological functions. Genistein is an isoflavone that binds to and inhibits protein-tyrosine kinase, resulting in increased intracellular cAMP levels, which leads to potentiation of CFTR activity in cell models (Illek and Fischer, 1998). However, clinical benefits were not clearly demonstrated when it was used in combination with 4-phenylbutyrate (NCT00590538). Other potentiators have been identified by cell-based HTSs but did not reach the clinical investigation for CF, such as the phenylglycine molecule PG-01 (Pedemonte et al., 2005b) and VRT-532 (Van Goor et al., 2006).

Ivacaftor (VX-770; Vertex Pharmaceuticals) is a potentiator identified by HTS that partially restored CFTR activity in G551D-expressing cell lines and in primary bronchial epithelial cells (Van Goor et al., 2009). Although the mechanisms of action of ivacaftor are not entirely elucidated, it was demonstrated to mediate CFTR channel potentiation in a phosphorylation-dependent and ATP-independent manner (Eckford et al., 2012; Cui et al., 2019). It binds and potentiates CFTR function by promoting decoupling between ATP hydrolysis and gating cycles (Jih and Hwang, 2013). Several studies have been proposing the putative binding site for ivacaftor by distinct methods. Some initial reports indicated that ivacaftor might bind to a region of amino acids at the NBD1/2 interface and the coupling helix of intracellular loop 1 (Veit et al., 2014) and/or the ‘ball-and-socket’ joint close to intracellular loop 4 (Byrnes et al., 2018). More recent studies provided direct evidence of the binding site of ivacaftor at the interface between TMDs by using electron cryomicroscopy and electrophysiological assays (Liu F. et al., 2019; Yeh et al., 2019). As chemical interactions are dynamic and the protein undergoes multiple conformational changes during channel opening and closure, some binding sites might also differ depending on the protein conformational state.

In 2012, both the U.S. Food and Drug Administration (FDA) and the European Medicines Agency (EMA) approved the use of ivacaftor (Kalydeco^®^, Vertex Pharmaceuticals) for CF patients aged ≥6 years carrying at least one G551D mutation after a phase III trial showing that ivacaftor has successfully improved clinical outcomes (sweat chloride concentration, percent predict forced expiratory volume in 1 sec [ppFEV_1_], and others) (Ramsey et al., 2011). A similar effectiveness was also observed in patients carrying one G551D mutation who had a more severe impairment in lung function (Barry et al., 2014). Ivacaftor is on the market for over 7 years, and it is transforming patients' lives with sustained and long-term benefits, including reduction in sweat chloride to normal levels, slower deterioration of lung function, reduction in the number of pulmonary exacerbation episodes, less frequent detection of *Pseudomonas aeruginosa* (McKone et al., 2014; Heltshe et al., 2015) and other common pathogens (Frost et al., 2019), better body mass index (BMI) (Borowitz et al., 2016), exercise capacity and well-being (Edgeworth et al., 2017), and quality of life (Quittner et al., 2015; Sawicki et al., 2015). Furthermore, observational studies have demonstrated that ivacaftor improved pancreatic function and mucociliary clearance. Reduction in the levels of blood inflammatory biomarkers, and smooth muscle and lung structure abnormalities have also been observed (Adam et al., 2016; Hisert et al., 2017; Donaldson et al., 2018b; Ronan et al., 2018).

A subsequent series of experimental and clinical studies led to the extended approval of ivacaftor to treat CF patients carrying other gating mutations (Yu et al., 2012; De Boeck et al., 2014). Thereafter, another extension has been approved to include CFTR mutations with residual function based on *in vitro* studies (Durmowicz et al., 2018), totalizing 38 eligible CF-causing mutations and increasing the number of patients who may benefit from ivacaftor treatment (**Table 3**). Notably, the membrane CFTR conductance depends on the number of functional channels, the average open probability and conductance of each CFTR channel (**Figure 5**). Therefore, some splicing mutations have shown to respond to ivacaftor treatment due to an improvement of the last two determinants, even though presenting less protein abundance at the PM. More recently, ivacaftor treatment has been extended for CF patients aged ≥6 months carrying at least one of the listed mutations (**Table 3**) (Davies et al., 2016; Rosenfeld et al., 2018; Rosenfeld et al., 2019). In an animal model of CF, intrauterine and postnatal administration of ivacaftor also demonstrated to protect ferrets (G551D/G551D genotype) against development of multi-organ disease (Sun et al., 2019), indicating that starting the treatment in newly diagnosed children may increase the chances of improving long-term outcomes or even preventing the appearance of certain symptoms. Long-term use of ivacaftor has also been associated with a decreased need for lung transplant and improved survival (Bessonova et al., 2018; Volkava et al., 2019). Despite all demonstrated benefits, patients receiving ivacaftor may eventually experience pulmonary exacerbations, thus requiring hospitalizations and worsening quality of life (Solem et al., 2016). Furthermore, ivacaftor treatment does not improve the rate of complete lung function recovery after an exacerbation episode (Flume et al., 2018). Lung function also declines in the long term, albeit at a slower rate.

**Table 3:** List of CF transmembrane conductance regulator (CFTR) mutations eligible for the treatment with Kalydeco^®^ and Symdeko^®^/Symkevi^®^.

**Kalydeco^®^**			
E56K	P67L		R74W
D110E	D110H		R117C
R117H	G178R		E193K
L206W	R347H		R352Q
A455E	S549N		S549R
G551D	G551S		D579G
711+3A>G	E831X		S945L
S977F	F1052V		K1060T
A1067T	G1069R		R1070Q
R1070W	F1074L		D1152H
G1244E	S1251N		S1255P
D1270N	G1349D		2789+5G>A
3272-26A>G		3849+10kbC>T	
**Symdeko^®^** or **Symkevi^®^ (‡)**			
F508del	P67L		D110H
R117C	L206W		R347H
R352Q	A455E		D570G
711+3A>G	E831X		S945L
S977F	R1070W		D1152H
2789+5G>A	3272-26A>G		3849+10kbC>T

(‡) Patients should carry at least one F508del mutation.

Several novel potentiators have demonstrated promising effects and are currently under experimental and clinical investigations (Phuan et al., 2015; Park et al., 2016; Yeh et al., 2017; Gees et al., 2018). The potentiators VX-561 (deutivacaftor or formerly CTP-656; Vertex Pharmaceuticals) is a deuterated form of ivacaftor that demonstrated an enhanced stability *in vitro* and in healthy volunteers compared to ivacaftor (Harbeson et al., 2017), which would allow CF patients to take it once daily rather than twice as it has been the case with the ivacaftor. A phase II trial is in progress to comparatively evaluate the effects of VX-561 and ivacaftor in CF patients who have the following CFTR gating mutations in at least one allele: G178R, S549N, S549R, G551D, G551S, G1244E, S1251N, S1255P, or G1349D (NCT03911713).

**Figure 7:**
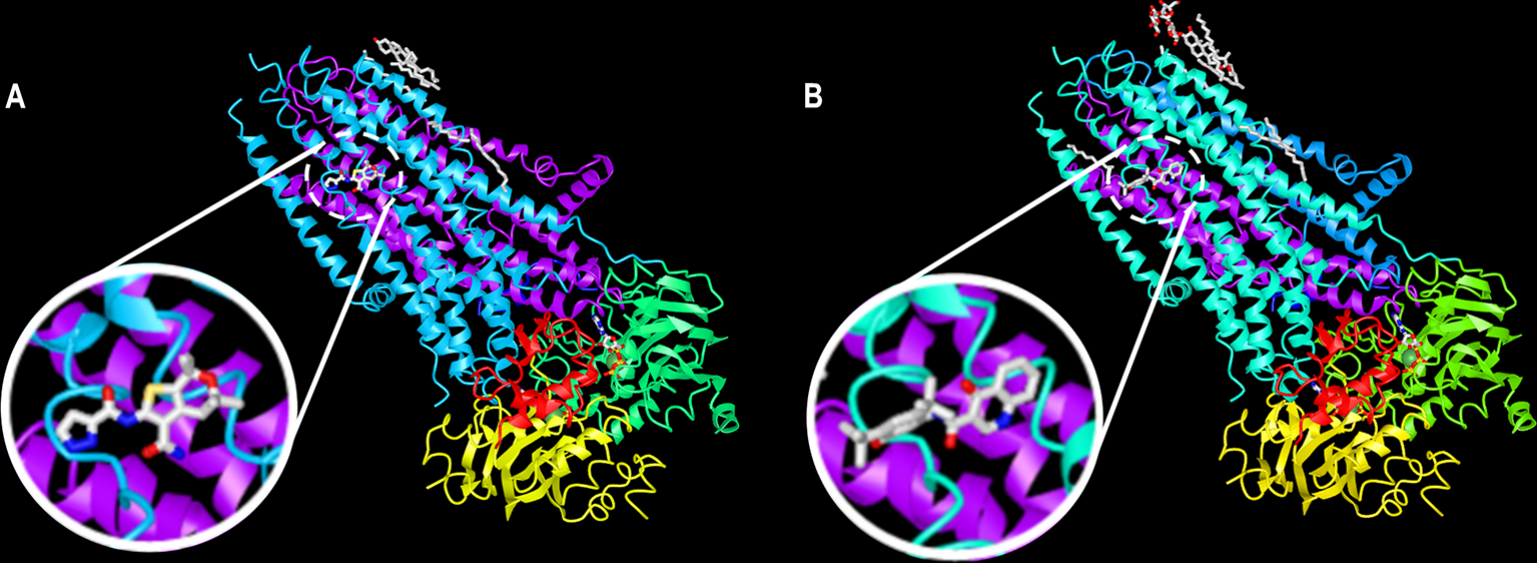
A common binding site for ABBV-974 and ivacaftor. Ribbon diagram of the phosphorylated, ATP-bound CFTR in complex with **(A)** ABBV-974 and **(B)** ivacaftor. These structures have been deposited on the Protein Data Bank under accession codes 6O1V (CFTR-ABBV-974) and 6O2P (CFTR-ivacaftor). (adapted from Liu F. et al., 2019 with permission from Prof. J. Chen).

The molecules ABBV-974, ABBV-2451, and ABBV-3067 (formerly GLPG-1837, GLPG-2451, and GLPG-3067, respectively) have been developed by Abbvie/Galapagos. ABBV-974 and ABBV-2451 have demonstrated greater and similar CFTR potentiation, respectively, in comparison with ivacaftor in R117H-, G178R-, S549N-, and G551D-expressing cells (Gees et al., 2018; Van der Plas et al., 2018). Interestingly, the simultaneous administration of ABBV-974 or ABBV-2451 with ivacaftor did not further enhance CFTR function, indicating that these molecules may act by the same mechanism of action (Yeh et al., 2017; Van der Plas et al., 2018). In fact, recent reports demonstrated that ABBV-974 and ivacaftor share a common binding site at the interface between the two TMDs near the kink of helix 8 (Liu F. et al., 2019; Yeh et al., 2019) (**Figure 7**). In a phase IIa trial, patients were subjected to a one-week ivacaftor washout before receiving three consecutive and increasing doses of ABBV-974. Although some adverse effects were observed in a dose-dependent manner, treatment was able to decrease sweat chloride concentration and improve ppFEV_1_ in CF patients carrying G551D-CFTR in at least one allele (Davies et al., 2019a).

The molecules FDL176 (Flatley Discovery Lab) and PTI-808 (dirocaftor; Proteostasis Therapeutics) are other potentiators being evaluated in early clinical trials (NCT03173573 and NCT03251092). The potentiator QBW251 (Novartis) was tested in both healthy volunteers and CF patients in a phase I/II clinical trial (NCT02190604). However, no significant improvements were observed in lung function of CF patients and adverse effects were also reported in many participants, resulting in early termination of this study. Recent studies have also demonstrated that co-administration of potentiators with complementary mechanisms of action may be an interesting approach for those CF-causing mutations in which CFTR gating/conductance is not completely restored by single potentiators (Phuan et al., 2018; Veit et al., 2019).

### Correctors: Rescuing the Protein Folding, Processing, and Trafficking

Most CF patients carry a mistrafficking CFTR mutation, since F508del is the most prevalent CF-causing mutation (Class II, **Figure 4**). Other common mutations that cause such abnormality are the G85E, I507del, R560T, and N1303K (found in 0.4%, 0.5%, 0.2%, and 1.6% CF alleles, respectively) (CFTR2 Database). Correctors are compounds that rescue folding, processing and trafficking to the PM of a CFTR mutant. While these compounds may act by distinct mechanisms, they usually enhance protein conformational stability during the ER folding process (Lopes-Pacheco, 2016).

New pharmacotherapies may target the defective CFTR structure directly by binding to the misfolding protein (termed as pharmacological chaperones) and/or indirectly by modulating its interaction with protein homeostasis (termed as proteostasis regulators), thus enabling the passage of CFTR protein through the ER quality control checkpoints (Lukacs and Verkman, 2012). Studies using genome- and proteome-wide association analyses have identified proteostasis components that could be targeted to rescue CFTR in F508del-expressing cells (Wang et al., 2006; Simpson et al., 2012; Tomati et al., 2018a). Furthermore, the recent identification of differences in WT- and F508del-CFTR interactomes unveiled several targets that could be exploited to rescue F508del (Pankow et al., 2015) and potentially other misfolding CFTR mutants (Hutt et al., 2018).

Initial studies were focused on certain proteostasis regulators, such as 4-phenylbutyrate (Rubenstein et al., 1997) and miglustat (Norez et al., 2006; Noël et al., 2008). Although these molecules demonstrated an increase in functional expression of F508del-CFTR in experimental models, only modest to no efficacy was found in early clinical studies (Zeitlin et al., 2002; Leonard et al., 2012). Several studies have also demonstrated a rescue of CFTR processing and trafficking to the PM in F508del-expressing cells by knocking down expression of certain proteostasis components, such as Aha1, an Hsp90 cochaperone (Wang et al., 2006), HDAC7 (Hutt et al., 2010), Hsp27 (Ahner et al., 2013; Lopes-Pacheco et al., 2015), and CFTR-associated ligand (CAL) (Bergbower et al., 2018), among others.

Advances in HTS technologies have been facilitating the fast-tracking of novel chemical compounds that act as proteostasis regulators and/or pharmacological chaperones (Pedemonte et al., 2005a; Van Goor et al., 2006; Noël et al., 2008; Kalid et al., 2010; Sampson et al., 2011). A classification of pharmacological chaperones has been proposed based on their molecular targets in the CFTR structure: class 1 correctors stabilize NBD1-TMD1 and/or NBD1-TMD2 interfaces; class 2 correctors stabilize NBD2 and its interfaces with other CFTR domains; and class 3 correctors directly stabilize NBD1 (Okiyoneda et al., 2013). This classification may be useful to evaluate corrector combinations with complementary mechanisms of action or targeting different defects in the mutant CFTR structure.

Lumacaftor (VX-809; Vertex Pharmaceuticals) is a first-generation corrector that demonstrated remarkable rescue of CFTR folding and function in F508del-expressing cell lines and primary bronchial epithelial cells (Van Goor et al., 2011). Other first-generation correctors had been identified previously but they were not suitable for the clinical practice due to a significant cytotoxicity and reduced efficacy (Pedemonte et al., 2005a; Van Goor et al., 2006). Some reports have suggested that lumacaftor has a putative binding site at the interface of NBD1 with the intracellular loop 4 in TMD2 (He et al., 2013; Okiyoneda et al., 2013; Hudson et al., 2017). Other studies have also indicated that lumacaftor acts on TMD1 and promote interactions between NBD1 and the intracellular loop 1 in TMD1 (Ren et al., 2013; Loo and Clarke, 2017; Laselva et al., 2018). While the identification of single putative binding site for small molecules, such as lumacaftor and ivacaftor, is important to unravel the mechanism(s) of action, multiple binding sites on the CFTR protein remain a possibility. Notably, lumacaftor was demonstrated to rescue ABCA4 trafficking mutants, another ABC transporter that shares approximately 45% homology with CFTR in NBDs (Sabirzhanova et al., 2015; Liu Q. et al., 2019).

Despite promising results *in vitro*, lumacaftor alone failed to demonstrate any improvement in lung function in F508del-homozygous patients in a phase IIa clinical study (Clancy et al., 2012). A significant, albeit modest, improvement in lung function (3%–4% in ppFEV_1_) was found only when lumacaftor was co-administered with ivacaftor in a following clinical trial (Boyle et al., 2014). Nevertheless, treatment with lumacaftor alone or in combination with ivacaftor was not able to improve ppFEV_1_ of F508del-heterozygous patients (Boyle et al., 2014; Rowe et al., 2017b). Certain adverse effects, such as chest tightness and dyspnea, have also been reported by both F508del-homozygous and -heterozygous patients in the early stage of the treatment.

In 2015, the FDA and the EMA approved the co-treatment with lumacaftor/ivacaftor (Orkambi^®^, Vertex Pharmaceuticals) for F508del-homozygous patients aged ≥12 years (Wainwright et al., 2015). Initial clinical trials usually tend to exclude patients with more severe disease, as they might present a more accelerated rate of decline in lung function (Habib et al., 2019). Nevertheless, F508del-homozygous patients with a more severe impairment of lung function were demonstrated to benefit from co-treatment initiation with lumacaftor/ivacaftor at a lower dose (Taylor-Cousar et al., 2019). Co-treatment with lumacaftor/ivacaftor was also demonstrated to benefit F508del-homozygous patients with distinct lung function impairment in a pooled analysis by subgrouping patients based on the FEV_1_ baseline (Elborn et al., 2016). Although the temporal process of recovery from pulmonary exacerbations may differ in each patient (Flume et al., 2019), co-treatment with lumacaftor/ivacaftor reduced the number of pulmonary exacerbation episodes even in patients without early lung function improvement (McColley et al., 2019). Furthermore, longer-term use of lumacaftor/ivacaftor has demonstrated continued benefits in patients, including improvement in BMI, reduction in the incidence of pulmonary exacerbations and hospitalizations, and slower deterioration of lung function (Konstan et al., 2017). Moreover, an observational study measured certain CFTR biomarkers — nasal potential differences and intestinal current measurements — and demonstrated that co-treatment with lumacaftor/ivacaftor results in partial correction of F508del-CFTR function to similar levels of the lower range of CFTR activity usually observed in patients with residual function mutations (Graeber et al., 2018). More recently, co-treatment with lumacaftor/ivacaftor has been extended for F508del-homozygous patients aged ≥2 years after phase III clinical trials demonstrating safety and efficacy in younger patients (Milla et al., 2017; Ratjen et al., 2017; McNamara et al., 2019). A clinical trial has also evaluated the effects of lumacaftor/ivacaftor in patients carrying A455E in at least one allele (NCT03061331), since this mutation has demonstrated an increase in CFTR PM abundance and function after corrector treatments in cell models (Dekkers et al., 2016a; Dekkers et al., 2016b; Lopes-Pacheco et al., 2016).

Lumacaftor has demonstrated different efficiency and potency in rescuing other CFTR mutations causing protein misfolding. In cell lines, lumacaftor was able to rescue CFTR function for E92K, L1077P, and M1101K, although it had no effect on G85E, R560S, and N1303K (Avramescu et al., 2017; Lopes-Pacheco et al., 2017; Awatade et al., 2019). The correction effects were also variable in patients-derived specimens carrying the F508del mutation in one allele and a minimal function mutation in *trans* (i.e., in the second allele); the mutants A561E, Y1092X, and W1282X demonstrated a response to lumacaftor treatment (Awatade et al., 2014; Haggie et al., 2017), but no effect was found for E60X, 394delTT, 711-1G>T, G542X, 1717-1G>A, and N1303K, among others (Awatade et al., 2014; Dekkers et al., 2016a; Pranke et al., 2017). Even for F508del-homozygous patients, co-treatment with lumacaftor/ivacaftor resulted in significant but variable clinical responsiveness (Boyle et al., 2014; Wainwright et al., 2015). The routine use of patient-specific biomarkers could be an effective approach to pre-clinically evaluate the safety and efficacy of certain pharmacotherapies and predict effectiveness at an individual level.

Notably, interaction between lumacaftor and ivacaftor revealed to negatively impact the rescue of F508del-CFTR protein. Chronic ivacaftor exposure (>1 µM) reduces lumacaftor-rescued CFTR in F508del-expressing cells (Cholon et al., 2014; Veit et al., 2014), although lower concentrations of ivacaftor (≤1 µM) may prevent such negative effect (Matthes et al., 2016). Lumacaftor also triggers cytochrome P450 3A4 activation, resulting in reduced plasma concentration of ivacaftor (Scheneider, 2018). Such findings might partially explain the modest efficacy observed in co-treatment with lumacaftor/ivacaftor in clinical trials. It also highlights the relevance of better evaluating drug-drug and drug-protein interactions for combined therapies. Recent studies have searched for potentiators of F508del-CFTR that do not interfere with lumacaftor actions (Phuan et al., 2015). Other studies have also identified genes of which either silencing or inhibition induce further lumacaftor-stimulated F508del-CFTR rescue, including RNF5 (Tomati et al., 2015; Sondo et al., 2018), FAU (Tomati et al., 2018a), and RFFL (Okiyoneda et al., 2018). Suppression of RPL12, a component of 60S subunit P stalk, was also demonstrated to rescue folding and function of F508del and other CFTR mutants by modulating ribosome velocity. CFTR rescue was increased when lumacaftor was concurrently administered (Veit et al., 2016b; Oliver et al., 2019).

Individuals with CF are susceptible to infection by distinct opportunistic pathogens, including *Aspergillus* spp., *Burkholderia cepacia*, *Haemophilus influenzae*, *Pseudomonas aeruginosa*, *Staphylococcus aureus*, and *Stenotrophomonas maltophilia*. Some studies have demonstrated that CFTR modulator therapies may reduce *P. aeruginosa* infection in clinics (McKone et al., 2014; Heltshe et al., 2015; Wainwright et al., 2015). Co-administration of lumacaftor/ivacaftor *in vitro* also reduced pro-inflammatory responses induced by *P. aeruginosa* exoproducts in well-differentiated human bronchial epithelial cells (F508del/F508del genotype) (Ruffin et al., 2018). Nevertheless, *P. aeruginosa* infection was demonstrated to reduce lumacaftor- and lumacaftor/ivacaftor-stimulated CFTR activity in F508del-expressing cells (Stanton et al., 2015). On the other hand, exposure of cells to supernatant from mucopurulent material derived human CF lung resulted in greater rescue of F508del-CFTR by lumacaftor (Gentzsch et al., 2018). As both inflammation and infection may influence responses to modulator therapies, further research is needed to better understand such differences as well as to evaluate the effect of these therapies on infection caused by other pathogens.

Tezacaftor (VX-661; Vertex Pharmaceuticals) is a second-generation corrector developed based on lumacaftor structure but demonstrating better pharmacokinetic properties and fewer adverse effects. In clinical trials, co-treatment with tezacaftor/ivacaftor demonstrated comparable therapeutic outcomes (sweat chloride concentration, ppFEV_1_, and others) to those with lumacaftor/ivacaftor in F508del-homozygous patients (Taylor-Cousar et al., 2017; Donaldson et al., 2018a). For F508del-heterozygous patients with a residual function mutation in *trans*, co-treatment with tezacaftor/ivacaftor was more effective, demonstrating even better improvements in ppFEV_1_ in comparison to treatment with ivacaftor only (Rowe et al., 2017a). In 2018, the FDA and the EMA approved the co-treatment with tezacaftor/ivacaftor (Symdeko^®^ or Symkevi^®^, Vertex Pharmaceuticals) for patients aged ≥12 years who are F508del-homozygous or F508del-heterozygous with a residual function mutation in *trans* (**Table 3**). In following phase III clinical trials, co-treatment of tezacaftor/ivacaftor was demonstrated to reduce sweat chloride concentration and preserve lung function with relatively low respiratory symptom burden (Walker et al., 2019). Such findings served as basis for the extended approval of tezacaftor/ivacaftor for patients aged ≥6 years. Extension clinical trials are ongoing to evaluate the longer-term effects of tezacaftor/ivacaftor (NCT03537651) and the safety and efficacy in younger children.

As the dual combinations lumacaftor/ivacaftor and tezacaftor/ivacaftor demonstrated only modest efficacy in F508del-homozygous patients, Vertex Pharmaceuticals performed additional HTSs to identify next-generation correctors that act by different mechanisms and could therefore yield additive/synergistic effects in triple-combination regimens. Tezacaftor/ivacaftor was selected as the backbone for the triple combination based on the more favorable pharmacological properties, including lower cytochrome P450 3A activation (Rowe et al., 2017a; Taylor-Cousar et al., 2017; Donaldson et al., 2018a). Four novel correctors — VX-152, VX-440, VX-445, and VX-659 — demonstrated a pronounced improvement of CFTR activity when co-administered with tezacaftor/ivacaftor in human bronchial epithelial cells (F508del/F508del genotype). In early-stage clinical studies, all four triple combinations presented evidence of therapeutic benefits to relatively similar degrees (NCT02951195, NCT02951182, NCT3029455, and NCT03227471). Nevertheless, two compounds (VX-445 [or elexacaftor] and VX-659 [or bamocaftor]) demonstrated more suitable pharmacological properties and safety profiles for long-term use, making them better candidates for subsequent clinical studies. Both triple combinations — tezacaftor/ivacaftor/VX-445 and tezacaftor/ivacaftor/VX-659 — were safe, with most adverse effects being mild to moderate, and led to a reduction of sweat chloride concentration and incidence of pulmonary exacerbations in phase II and III trials. A significant increase in ppFEV_1_ was also found after triple-combination regimens in F508del-homozygous patients (up to 11.0% for the combination with VX-445 and 9.7% for the combination with VX-659 compared to tezacaftor/ivacaftor only) and F508del-heterozygous patients with a minimal function mutation in *trans* (up to 14.3% for the combination with VX-445 and 13.3% for the combination with VX-659 compared to placebo) (Davies et al., 2018; Keating et al., 2018; Heijerman et al., 2019; Middleton et al., 2019; Taylor-Cousar et al., 2019). In fact, the magnitude of therapeutic responses with triple-combination regimens was even greater than the benchmark achieved by ivacaftor alone in patients with a G551D-CFTR mutation (up to 10.6% in ppFEV_1_) (Ramsey et al., 2011; McKone et al., 2014; Donaldson et al., 2018a) or other gating mutations (De Boeck et al., 2014). The triple combination tezacaftor/ivacaftor/VX-445 (Trikafta™, Vertex Pharmaceuticals) has been recently approved by the FDA for the treatment of CF patients aged ≥12 years with the mutation F508del in at least one allele. Furthermore, other clinical trials are in progress to evaluate the long-term effects of this triple combination in patients who are F508del-homozygous or F508del-heterozygous with a minimal function mutation in *trans* (NCT03525574, NCT04043806, NCT04058366) as well as the safety and efficacy in younger patients (NCT03691779, NCT04183790). Another clinical study is also ongoing to evaluate the safety and efficacy of this triple-combination regimen in F508del-heterozygous patients with a gating or residual function mutation in *trans* (NCT04058353).

Other compound combinations have been evaluated in order to enhance the correction of F508del-CFTR, but only modest effects were observed in several experimental studies (Okiyoneda et al., 2013; Phuan et al., 2014; Lopes-Pacheco et al., 2015). A rational design for combinatory corrector therapy resulted in the discovery of small-molecule from different chemical series — 6258, 3151, and 4172 — that target defects at NBD1, NBD2, and TMD interfaces. Although the individual administration of these compounds was demonstrated to modestly rescue the functional expression of F508del-CFTR, the combination of three compounds resulted in greater effects than lumacaftor alone. The effects of this triple combination on F508del-CFTR reached ~50-100% of WT-level correction in cell lines, patient-derived specimens, and in mouse nasal epithelia. Moreover, this triple combination significantly increased protein folding efficacy of rare mutations across different CFTR domains (Veit et al., 2018).

Several novel correctors are being developed in collaboration between pharmaceutical companies and academic laboratories. The molecules FDL169 and FLD1737 have been investigated by Flatley Discovery Lab. FDL169 has demonstrated a rescue of CFTR PM expression in F508del-expressing cells with similar efficacy as lumacaftor but with no additive effects when they were co-administered, suggesting that these correctors may act by the same mechanisms. Nevertheless, ivacaftor had lesser inhibitory effect on FDL169 activity than on lumacaftor. The safety profile of FDL169 is under clinical investigation alone and in combination with the potentiator FDL176 (NCT02768297, NCT03093714, NCT03756922). Riociguat (Bayer), a soluble guanylate cyclase stimulator, was demonstrated to improve CFTR function in experimental studies. Although no safety concerns were identified in F508del-homozygous patients in a phase II trial (NCT02170025), the clinical development of this molecule has been terminated for CF.

The molecules ABBV-2222, ABBV-2737, ABBV-2851, ABBV-3221, and ABBV-3748 (formerly GLPG-2222, GLPG-2737, GLPG-2851, GLPG-3221, and GLPG-3748, respectively) are among the most promising correctors developed by Abbvie/Galapagos. ABBV-2222 (or galicaftor) has a similar chemical structure to lumacaftor and tezacaftor but was reported to be more potent. It was also highly functional in primary bronchial epithelial cells from a F508del-homozygous patient (Wang et al., 2018; Singh et al., 2019). ABBV-2737 was demonstrated to rescue functional expression of CFTR in F508del-expressing cells, and such effects were enhanced when it was co-administered with lumacaftor or ABBV-2222 (de Wilde et al., 2019). ABBV-3221 also rescued CFTR function in F508del-expressing cells with a greater effect in combination with ABBV-2222 and ABBV-974 (Scanio et al., 2019). In phase IIa clinical trials, ABBV-2222 reduced sweat chloride concentrations but did not improve ppFEV_1_ in F508del-homozygous patients or F508del-heterozygous patients with a gating mutation and receiving ivacaftor (Bell et al., 2019). Based on previous studies evaluating drugs with a similar mechanism of action in F508del-homozygous patients (Clancy et al., 2012; Boyle et al., 2014; Wainwright et al., 2015), a substantial improvement in lung function would be unusual for a single corrector. Nevertheless, such approach allows a better evaluation of the safety profile for the following studies with combined therapies. In this line, other clinical studies are in progress to evaluate the safety and efficacy of ABBV-2222 alone and in combination with ABBV-2737 and ABBV-2451 (NCT03540524) or with ABBV-3067 (NCT03969888). ABBV-2737 was also investigated for F508del-homozygous patients and on stable treatment with lumacaftor/ivacaftor in a phase IIa trial. A small improvement in ppFEV_1_ (3.6%) and a significant reduction in sweat chloride concentration were found in patients that received ABBV-2737 compared to placebo cohort (van Koningsbruggen-Rietschel et al., 2019).

The molecule PTI-801 (posenacaftor; Proteostasis Therapeutics) — a third-generation corrector — has demonstrated higher efficacy compared to first- and second-generation correctors (e.g., lumacaftor and tezacaftor) with additive/synergistic effects when they were co-administered *in vitro*. PTI-801 was also less sensitive to the ivacaftor-mediated decrease on CFTR function. In a phase I trial, PTI-801 was demonstrated to significantly reduce sweat chloride concentration, and improve ppFEV_1_ and BMI in F508del-homozygous patients receiving lumacaftor/ivacaftor therapy. Another phase I clinical trial is in progress in healthy volunteers and CF patients (NCT03140527). The corrector VX-121 (Vertex Pharmaceuticals) is also in early-stage clinical trials to evaluate the effects in CF patients receiving ivacaftor or tezacaftor/ivacaftor (NCT03768089) and in combination with tezacaftor, ivacaftor, and/or VX-561 (NCT03912233).

### Stabilizers: Rescuing the Protein Stability at the Plasma Membrane

Although certain CFTR mutants are functional and present at the PM, the protein may still display a significant reduction in half-life (Haardt et al., 1999) (Class VI, **Figure 4**), probably due to accelerated endocytosis (Swiatecka-Urban et al., 2005) and/or reduced recycling (Sharma et al., 2004). Low temperature incubation rescues CFTR in F508del-expressing cells (r.F508del) (Denning et al., 1992), but the protein still demonstrates reduced stability and is rapidly removed by peripheral quality control mechanisms (Okiyoneda et al., 2010). In fact, environmental stresses may also lead to destabilization and internalization of WT-CFTR (Bomberger et al., 2012; Patel et al., 2019). Stabilizers are agents that anchor CFTR at the PM, thus preventing its removal and degradation by lysosomes (Fukuda and Okiyoneda, 2018).

Lumacaftor demonstrated to rescue functional expression of CFTR in F508del-expressing cells, but it does not confer long-term stability similar of that in WT-CFTR (He et al., 2013). Therefore, novel treatments have been investigated, alone or in combination, to rectify the intrinsic protein instability and thus prolong CFTR residence time at the PM (Fukuda and Okiyoneda, 2018). Hepatocyte growth factor (HGF) was demonstrated to promote CFTR stabilization at the PM in F508del-expressing cells by activating Rac1 GTPase signaling and thus inducing CFTR interaction with Na^+^/H^+^ exchanger regulatory factor 1 (NHERF1) (Moniz et al., 2013). Co-administration of HGF and lumacaftor has further enhanced CFTR maturation and anchoring at the PM (Loureiro et al., 2015). Prolonged HGF treatment also prevented ivacaftor-mediated destabilization of lumacaftor-rescued CFTR in F508del-expressing cells (Matos et al., 2018). Other strategies have demonstrated to enhance the PM stabilization of mutant CFTR protein, including administration of vasoactive intestine peptide (VIP) (Alshafie et al., 2014), activation of exchange factor directly activated by cAMP 1 (EPAC1) (Lobo et al., 2016), and inhibition of S-nitrosoglutathione reductase (Zaman et al., 2016) or CFTR-associated ligand (CAL) (Cusing et al., 2010; Bergbower et al., 2018). More recently, keratin-19 was also demonstrated to stabilize WT-CFTR and lumacaftor-rescued F508del at the PM by decreasing Rab7-mediated lysosomal degradation (Hou et al., 2019).

Cavosonstat (N91115; Nivalis) was the first CFTR stabilizer being tested in clinical trials. It was demonstrated to promote CFTR maturation and PM stability by inhibiting S-nitrosoglutathione reductase *in vitro*. In a phase I trial, cavosonstat demonstrated no safety concerns with the highest dose yet reducing sweat chloride concentration in F508del-homozygous patients (Donaldson et al., 2017). Nevertheless, cavosonstat failed to demonstrate any additional benefit in lung function and sweat chloride concentration when in combination with lumacaftor/ivacaftor or ivacaftor in phase II trials (NCT02589236 and NCT02724527). The clinical development of cavosonstat has been terminated for CF.

### Read-Through Agents and NMD Inhibitors: Rescuing the Protein Synthesis

A significant fraction of the CF-causing mutations are in-frame nonsense, frameshift, and splicing variants that introduce a premature termination codon (PTC) into the CFTR mRNA, thus abrogating CFTR protein synthesis or resulting in translation of shortened, truncated forms (Class I, **Figure 4**). PTCs are also subjected to nonsense-mediated mRNA decay (NMD), resulting in substantial decrease in the quantity of CFTR transcripts (Nguyen et al., 2014; Sharma N. et al., 2018). Around 10% of CF patients worldwide carry a PTC mutation, with G542X and W1282X being the most common PTC variants found in CF alleles (2.5% and 1.2%, respectively) (CFTR2 Database). Read-through agents are compounds that induce a ribosomal “over-reading” of a PTC, enabling the incorporation of a foreign amino acid in that place and thus the continued translation to the normal end of the transcript (Pranke et al., 2018).

Read-through effects were first found in aminoglycoside antibiotics, such as gentamicin and geneticin. Gentamicin is also commonly used to eradicate *P. aeruginosa* infection in CF patients. In both cell lines and transgenic mice, these compounds demonstrated the ability to promote expression of full-length CFTR and partially restore CFTR-dependent chloride secretion (Howard et al., 1996; Du et al., 2002). Furthermore, gentamicin improved nasal potential difference after administration either topically on the nasal mucosa (Wilschanski et al., 2003) or intravenously (Sermet-Gaudelus et al., 2007) in patients carrying a CFTR PTC mutation. Despite such findings, gentamicin and geneticin cannot be used as read-through agents in the clinics, since high systemic levels or long-term use may result in severe nephrotoxicity and ototoxicity (Prayle et al., 2010). Advances in the rational design of chemically modified aminoglycosides have enabled the identification of read-through agents with higher activity and less toxicity (Rowe et al., 2011; Kandasamy et al., 2012). In this line, ELX-02 (NB124; Eloxx Pharmaceuticals) has demonstrated to restore CFTR function in cells expressing any of the four most prevalent PTC mutations — G542X, R553X, R1162X, and W1282X. Moreover, ELX-02 was less cytotoxic than gentamicin in a model for ototoxicity (Xue et al., 2014). In phase I clinical trials with healthy volunteers, ELX-02 was well tolerated and exhibited a favorable safety profile, although mild side effects were also reported (Leubitz et al., 2019). Early-stage clinical trials are in progress to evaluate the effects of multiple dose escalation of ELX-02 in CF patients carrying the G542X mutation in at least one allele (NCT04126473, NCT04135495).

Ataluren (PTC124; PTC Therapeutics) was identified by HTS (Welch et al., 2007), and demonstrated to restore CFTR expression and function in transgenic mice expressing human G542X (Du et al., 2008). Despite therapeutic effects observed in phase II clinical trials (Sermet-Gaudelus et al., 2010; Wilschanski et al., 2011), ataluren treatment only demonstrated a favorable trend, albeit not significant, to improve ppFEV_1_ of CF patients carrying a nonsense CFTR mutation in at least one allele in a phase III clinical trial (Kerem et al., 2014). A new phase III trial was undertaken to exclude patients taking inhaled tobramycin, since it could interfere with ataluren actions on the ribosome; however, no improvements were observed in ppFEV_1_ and pulmonary exacerbations (NCT02139306). Although ataluren (Translarna^®^, PTC Therapeutics) is approved for the treatment of patients with Duchenne muscular dystrophy, its clinical development has been terminated for CF. Other studies have performed HTSs to identify potential read-through agents for the various PTC mutations (Mutyam et al., 2016; Liang et al., 2017).

In addition to read-through agent-induced efficacy, the abundance of CFTR transcripts and the activity of the recoded protein are other factors that should be considered in order to efficiently rescue a PTC mutant (Pranke et al., 2018). The number of transcripts may considerably differ depending on the identity (amber, ochre, or opal) and position of the termination codon as well as cell type and patient's genetic background (Nguyen et al., 2014; Pranke et al., 2018; Sharma N. et al., 2018; Clarke et al., 2019), and association of an NMD inhibitor with a read-through agent may result in better therapeutic efficacy. In this line, amlexanox (Gonzalez-Hilarion et al., 2012) and escin (Mutyam et al., 2016) are drugs already approved for unrelated diseases that demonstrated dual activity by concomitantly increasing the abundance of target transcripts and read-through efficacy for certain CFTR PTC mutations. Furthermore, incorporation of a foreign amino acid may result in full-length but misfolded and/or nonfunctional proteins. PTC suppression in combination with other modulators, such as lumacaftor and/or ivacaftor, has demonstrated to promote a further rescue of expression and function of CFTR PTC mutations (Xue et al., 2014; Mutyam et al., 2016; Mutyam et al., 2017; Xue et al., 2017; Pranke et al., 2018; Sharma N. et al., 2018). These approaches should be exploited in future clinical studies.

A nucleic acid therapeutic has been developed for CF-causing nonsense mutations. RCT101 (ReCode Therapeutics) is a modified transfer RNA (tRNA) that is delivered into cells by nanoparticles to precisely recode the translating CFTR protein. In experimental studies, RCT101 demonstrated an increase in CFTR-dependent chloride secretion in primary bronchial epithelial cells carrying either G542X/G542X or G542X/F508del. Such effects were even greater when combined with lumacaftor/ivacaftor.

### Amplifiers: Increasing the Abundance of Protein Substrate

Some CF-causing mutations lead to reduction in the synthesis or maturation of CFTR protein. The 3849+10kbC>T, 2789+5G>A, and A455E are common mutations that cause such abnormalities (Class V, **Figure 4**) and are found in 0.8%, 0.7%, and 0.4% of the CF alleles, respectively (CFTR2 Database). Amplifiers are compounds that increase expression of CFTR mRNA and, consequently, biosynthesis of the CFTR protein (Giuliano et al., 2018).

The PTI-428 (nesolicaftor; Proteostasis Therapeutics) is the first-in-class amplifier investigated in clinical trials. It was demonstrated to selectively increase the expression of immature CFTR protein carrying different mutations without eliciting alteration in the expression of cellular stress response genes (Giuliano et al., 2018). PTI-428 also enhanced the rescue of CFTR in F508del- and ΔI1234_R1239-expressing cells when co-administered with lumacaftor/ivacaftor (Molinski et al., 2017; Giuliano et al., 2018). Furthermore, the triple combination PTI-428/PTI-808/PTI-801 enhanced the CFTR-dependent chloride secretion to almost normal levels in F508del-expressing cells. In phase I/II clinical trials (NCT03500263), this triple combination regimen resulted in significant reduction of sweat chloride concentration and improvement of lung function (8% in ppFEV_1_) compared to placebo in F508del-homozygous patients, being the greatest effects observed in those individuals with high disease burden. Based on these results, a phase III trial is planned to begin in early 2020. In F508del-heterozygous patients, the triple combination PTI-428/PTI-808/PTI-801 demonstrated a more variable change in sweat chloride concentration and lung function. These modulator drugs have also been tested in intestinal organoids of patients carrying rare CF genotypes in the HIT-CF project and the crossover clinical trial based on the individual responses is expected to initiate in the middle of 2020. Other early-stage clinical trials are in progress to evaluate the safety and efficacy of PTI-428 in CF patients on stable treatment with ivacaftor (NCT03258424), lumacaftor/ivacaftor (NCT02718495), or tezacaftor/ivacaftor (NCT03591094).

### Antisense Oligonucleotides: Correcting the Aberrant Splicing and More

Antisense oligonucleotides (ASOs) are chemically-modified synthetic RNA-like molecules that act by complementary base pairing to the target sequence. ASOs have been demonstrating promising results to correct nonsense and splicing mutations or even to replace missing bases caused by deletion mutations, such as the F508del.

ASOs corrected the aberrant splicing in a cell line expressing the 2789+5G>A mutation minigene. Such correction resulted in recovery of CFTR protein levels at the PM (Igreja et al., 2016). Recently, ASOs developed by SpliSense have also demonstrated to correct aberrant splicing and restore CFTR function in a 3849+10kbC>T-expressing cell line and in primary bronchial epithelial cells (3849+10kbC>T/F508del genotype).

The mutant W1282X is subjected to NMD, thus resulting in significant reduction of CFTR mRNA abundance. Nevertheless, it still produces a certain amount of the truncated forms of both partially and fully glycosylated CFTR protein that may respond to CFTR modulators (Haggie et al., 2017; Aksit et al., 2019). ASOs designed to downregulate the serine/threonine-protein kinase SMG-1, a factor involved in the NMD pathway, led to upregulation of mRNA, protein maturation and traffic to the PM of the truncated CFTR products in W1282X-expressing cells. Furthermore, these ASOs increased CFTR-dependent chloride secretion in W1282X-homozygous cells (Keenan et al., 2019).

Eluforsen (QR-010; ProQR) is a modified RNA oligonucleotide that was demonstrated to restore CFTR function in a F508del-expressing cell line, patient-derived specimens and murine models (Beumer et al., 2019). Furthermore, eluforsen was able to efficiently diffuse through CF-like mucus layer on air-liquid interface cell cultures (Brinks et al., 2019). In an early-stage trial assessing single and multiple doses in F508del-homozygous patients, eluforsen was well tolerated and improved quality of life. Lung function also remained stable throughout the study (Drevinek et al., 2019). In a following clinical study, repeated intranasal administration of eluforsen resulted in improvement in the nasal potential difference in F508del-homozygous patients, but not in the F508del-heterozygous cohort (Sermet-Gaudelus et al., 2019). Although no severe safety concerns have been reported, the clinical development of eluforsen has been discontinued.

## Barriers and Future Directions for Precision Medicine to Reach All Individuals With CF

CFTR modulators have become transformative therapeutic approaches for many CF patients, as mentioned above. Despite several breakthroughs, further research is needed to continue optimizing therapies and to identify novel modulators for patients carrying rare, ultra-rare, or even unique CFTR mutations, who still face an unmet need for efficient, corrective therapies. Furthermore, some barriers still pose substantial challenges in the equitable availability of these pharmacotherapies, including the excessive costs and regulatory national issues. The collaborative environment composed by academic researchers, healthcare professionals, pharmaceutical companies, and patient representatives has been crucial in developing better treatments for people with CF.

### Continuing the Optimization of Therapeutic Regimens to Increase the Adherence and Reduce the Burden

The multifaceted nature of CF requires complex and time-consuming therapeutic regimens that should be periodically adapted according to disease progression. Furthermore, CF patients are subjected to substantial clinical, psychosocial, and economic burdens, which pose challenges to achieve optimal, lifelong treatment adherence. The adherence varies largely depending on treatment type, route of administration, duration, and number of distinct medications, as well as patient age and socioeconomic status (Sawicki et al., 2013; Angelis et al., 2015; Quittner et al., 2016; Narayanan et al., 2017). Moreover, CF patients usually spend twice and 20 times more time in daily treatment activities than diabetic and asthmatic patients, respectively (Ziaian et al., 2006), which may considerably affect adherence. Poor adherence has also been associated with higher healthcare costs, more frequent hospitalizations, and worse quality of life and clinical manifestations (Sawicki et al., 2013; Quittner et al., 2016; Narayanan et al., 2017). Establishing a closer relationship among patient, families/caregivers and the multidisciplinary healthcare team may be a first step to overcome key barriers to treatment adherence.

To date, only few publications have evaluated the adherence to ivacaftor treatment and adherence to modulator combinations remains yet to be demonstrated. From a clinical perspective, a life-transforming oral medication with a simple dosing schedule would supposedly be taken as prescribed. Adherence to ivacaftor has nevertheless varied from suboptimal (Siracusa et al., 2015) to optimal (Suthoff et al., 2016). As these studies had a small sample size and applied distinct methods, it is still difficult to extrapolate the results to a broader CF population, and further studies are needed to better address this issue. Certain therapeutic benefits may also be more modest in a real-world setting compared to clinical trials, as patients should take these oral medications following specific recommendations, including dietary to ensure better drug absorption and availability in the body. Furthermore, recent studies have demonstrated that abrupt interruption of CFTR modulator therapy may cause severe clinical consequences. Ivacaftor withdrawal resulted in accelerated deterioration of lung function consistent with a pulmonary exacerbation episode in a case series (Trimble and Donaldson, 2018). Patients who discontinued treatment with lumacaftor/ivacaftor, mainly due to early adverse effects, also demonstrated a higher risk of worsening clinical manifestations compared to patients who continued treatment or those who restarted it after temporary discontinuation in a real-world study (Burgel et al., 2020). As patients have different lifestyles and socioeconomic conditions, the development of educational and motivational interventions at an individual level may help in ensuring optimal adherence to achieve the greatest clinical outcomes.

CFTR modulators have been added to therapeutic regimens of eligible patients, rather than replacing some symptomatic therapies. This appears to be the optimal approach for most CF patients, although it also increases the burden of medications in use. Once the safety and efficacy of novel therapies are demonstrated in adults, extension clinical trials are pursued to evaluate the effects on younger patients, as adverse effects may vary across distinct age groups (Davies et al., 2016; Rosenfeld et al., 2018; McNamara et al., 2019; Rosenfeld et al., 2019). Starting these transformative therapies in milder disease severity and earlier in life may offer more chances of significantly improving long-term outcomes or even preventing certain injury of affected organs, which may also result in a lower burden of medications in a long-term perspective. Some reports have also demonstrated that younger patients are more adherent to therapies than adolescents and adults, possibly due to higher parental supervision (Quittner et al., 2014; Shakkottai et al., 2014). Providing educational and supporting approaches to young children and their parents may result in optimal, lifelong treatment adherence. As patients should be transferred from pediatric to adult care at a certain age (generally between 18 and 21 years old), a planned transition is greatly helpful to maximize independence, minimize chances of interruption in the therapies and continue improving their quality of life (Goralski et al., 2017).

### Continuing the Development of Transformative Therapeutics to Reach All Individuals With CF

Most development programs of CFTR modulators has been initially focused on the correction of F508del mutation, since fully overcoming the defects in this mutation would result in an effective therapy for approximately 82% of the CF patient population worldwide. There are still nevertheless 10%–18% of patients without any CFTR-directed therapeutics. This percentage is even higher in countries where the prevalence of F508del is much lower, such as Brazil, Israel, Italy and Turkey (**Figure 3**).

Identifying the putative binding sites of CFTR-directed modulators using the novel insights of CFTR structure may facilitate the rational design of novel compounds with enhanced pharmacological properties. The pipeline of CFTR modulators continues to expand and some recent drug development programs have also been pursuing the identification of modulators to less common CF-causing mutations. Identification of specific therapies for rare and ultra-rare mutations poses nevertheless several challenges due to the great variability of CF-causing mutations and the very small number of patients. In addition to CFTR-directed modulators, CFTR dysfunction might be compensated by targeting alternative ion channels, such as ENaC (Moore and Tarran, 2018), the calcium-activated chloride channel transmembrane protein membrane 16A (TMEM16A) (Sondo et al., 2014), and the solute carrier 26A9 (SLC26A9) (Balázs and Mall, 2018). Strategies that modulate these alternative ion channels might be efficient therapies for all patients, regardless of their CF genotypes. These strategies might also be used alone or in combination with CFTR modulators to enhance clinical outcomes. Nevertheless, as CF patients are already subjected to a substantial burden of medications, drug-drug interaction profiles should be further exploited to avoid adverse effects or inhibitory effects of one therapy on another. In this line, itraconazole, an antifungal commonly used for the treatment of allergic bronchopulmonary aspergillosis, was demonstrated to significantly increase systemic exposures of tezacaftor and ivacaftor (Garg et al., 2019). Caution and appropriate monitoring are recommended when these therapies are used at the same period.

Traditional trials with a placebo-controlled design have been providing evidence for the safety and efficacy of CFTR modulators (Habib et al., 2019) (**Table 1**); however, alternatives will be needed in the near future, as more modulator options become available and the number of patients without any modulator therapy will certainly reduce. Furthermore, clinical trials in sicker or younger patients, and those carrying rarer CFTR mutations are more challenging due to small sample size, specific inclusion/exclusion criteria, or even for some hesitation on the part of the investigators. Strategies to adapt and optimize trial design and deliver for speed and efficacy have been discussed, including the use of patient-derived specimens, power calculations to compensate for group sampling, and N-of-1 and “basket” trials (Matthes et al., 2018; Amaral et al., 2019; Davies et al., 2019b).

The use of patient-derived specimens to comparatively evaluate drug efficacies may be a feasible starting point to identify the best candidate drug(s) *in vitro* and predict the magnitude of therapeutic responses for following clinical testing (Strauss and Blinova, 2017; Amaral et al., 2019). In fact, a significant but variable clinical responsiveness was observed in clinical trials with CFTR modulators in patients carrying at least one G551D mutation (Ramsey et al., 2011; Rowe et al., 2014) or in F508del-homozygous patients (Boyle et al., 2014; Wainwright et al., 2015; Donaldson et al., 2018a), which suggests that patient responsiveness to a certain therapy is influenced not only by the CF genotype but also by the genetic background and/or epigenetic factors. In this line, some reports have demonstrated that single nucleotide polymorphisms in *SLC26A9* gene contribute to heterogeneity in inter-individual responsiveness to CFTR modulator therapies (Strug et al., 2016; Corvol et al., 2018). Such findings denote the relevance of assessing the drug effectiveness at an individual level in patient-derived specimens.

A report pairing *in vitro* measurement of CFTR function in cell lines and clinical features demonstrated a strong correlation between CFTR function and sweat chloride concentration, and to a lesser extent but still significant with lung function and pancreatic status (McCague et al., 2019). Correlations between responses in patient-derived specimens and clinical parameters/biomarkers have been investigated to establish reliable prediction of drug effectiveness. A consistent correlation was found among forskolin-induced swelling of intestinal organoids, sweat chloride concentration and intestinal current measurements of infants with CF (de Winter-de Groot et al., 2018). Despite the clinical heterogeneity in adults with CF and homozygous for F508del mutation, forskolin-induced swelling of intestinal organoids positively correlated with FEV_1_ and BMI (de Winter-de Groot et al., 2019). Responses from intestinal organoids were also demonstrated to correlate with intestinal current measurements, reduction in sweat chloride concentration and improvement in lung function of patients after CFTR modulator therapies (Dekkers et al., 2016a; Berkers et al., 2019). In N-of-1 trial series, an increase in CFTR-dependent chloride transport in nasal epithelial cell cultures was only found in the three patients who also demonstrated a reduction in sweat chloride concentration after ivacaftor treatment (McGarry et al., 2017). Furthermore, responses in F508del-homozygous patient-derived nasal epithelial cells were correlated to improvements in ppFEV_1_ and intestinal current measurements, but not with nasal potential difference after co-treatment with lumacaftor/ivacaftor (Pranke et al., 2019). Nevertheless, no significant correlations were found among responses in intestinal current measurement, nasal potential difference and sweat chloride concentration, despite high concordance for all CFTR-dependent biomarkers in another study evaluating the co-treatment with lumacaftor/ivacaftor (Graeber et al., 2018). Further studies are certainly needed to better correlate and validate drug effectiveness in patient-derived specimens with clinical features, and identification of novel biomarkers may also enrich strategies in efficacy trials.

In an era of drugs targeting the underlying defects in CF-causing mutations, the development of symptomatic therapies might appear less attractive. Nevertheless, these therapies must continue to be developed as most (if not all) existing CF population will need them at some point, and CFTR modulators are very unlikely to reverse lung tissue remodeling already established (Davies et al., 2019b). A recent study demonstrated that six months of ivacaftor treatment was unable to significantly change airway microbiome and several inflammation measurements in patients carrying at least one G551D mutation. Such findings indicate that antibiotics and anti-inflammatory drugs will still be required to control disease symptoms and prevent complications (Harris et al., 2019). As the disease progresses, patients may also develop comorbidities and thus require even more complex therapeutic regimens, adding further burdens. Ivacaftor treatment was demonstrated to improve exocrine pancreatic function as well as insulin secretion profile, which may alleviate or even reverse CF-related diabetes (Hayes et al., 2014; Davies et al., 2016; Tsabari et al., 2016; Kelly et al., 2019). Ivacaftor treatment was also demonstrated to improve bone health (Sermet-Gaudelus et al., 2016) and vascular tone abnormalities (Adam et al., 2016). A recent review nicely summarizes the current understanding of CFTR modulators on extra-pulmonary complications in CF (Sergeev et al., 2019). Nevertheless, most studies are case reports or have a small sample size, and further studies are warranted to investigate the impact of CFTR modulator therapies on CF comorbidities.

Treatment with more than one CFTR modulator appears to be the optimal approach for many CF-causing mutations. As heterozygous carriers are asymptomatic, fully overcoming CFTR dysfunction in one allele might be enough to halt disease progression, if treatment is started early in life and before severe lung injury occurs. Based on *in vitro* evidence (Zhang et al., 2009), rescue of 25-50% of WT-CFTR function in both alleles might also be sufficient to restore normal rates of mucociliary clearance. It remains nevertheless unclear how many CFTR modulators would be needed to reach such threshold in patients. In addition to CFTR modulators, progress has been made in developing cell-based (Barical et al., 2019; Hayes et al., 2019) and gene-based therapies (Donnelley and Parsons, 2018; Duncan et al., 2018; Lopes-Pacheco et al., 2018; Osman et al., 2018) for CF lung disease.

### Identifying Feasible Solutions for a CF Healthcare Cost Sustainable

A major limitation of these novel pharmaceutical treatments for CF patients, such as the CFTR modulators, is the excessive costs when they reach the market (over US$250,000 per patient per year), which renders difficulties in their availability for many patients worldwide (O'Sullivan et al., 2013; Ferkol and Quinton, 2015; Orestein et al., 2015), especially for those living in low- and middle-income countries (Cohen-Cymberknoh et al., 2016). In developed countries, certain health authorities have also been slow in approving reimbursement (Bush and Simmonds, 2012; Whiting et al., 2014; Sharma D. et al., 2018) and the cost-effectiveness of these pharmacotherapies has yet been questioned (Gulland, 2016; Balk et al., 2018). Even though the quality-adjusted life-year (QALY) analysis might not adequately address all concerns for rare diseases, such as CF (Schlander et al., 2014; Pearson et al., 2018), these therapies pose a substantial burden on national healthcare systems, as they are expensive and lifelong. It remains nevertheless unclear if such prices will persist over time, as several novel molecules are on the horizon and probably will reach the market over the next years, if they prove to be safe and to have efficacy in clinical studies. Further discussion should certainly be undertaken with patient representatives, healthcare providers, policymakers, government authorities and pharmaceutical companies to identify feasible and sustainable solutions that would enable equitable access to eligible patients for these “on-target” therapies. Hopefully, market competition will also reduce modulator prices with the approval of novel ones.

Over the past three decades, the human disease target landscape considerably expanded, as approximately 40% of approved pharmaceuticals received an orphan designation (Attwood et al., 2018). Drug discovery and development for a new molecule may be nevertheless far slower than expected, as it is a costly process with high attrition rates that also depends on several regulatory requirements. Drug repurposing (also known as drug repositioning or reprofiling) has become an increasingly attractive strategy that may save valuable time and funding investments in drug development for common and rare diseases. As approved drugs have already undergone extensive toxicological evaluations in both experimental and early-stage clinical studies, the time frame to obtain a new disease indication may be reduced, if safety and efficacy is demonstrated for the repurposed use in late-stage clinical studies (Pushpakom et al., 2019). Furthermore, drug repurposing may unravel effective therapies for patients with common and rare CF-causing mutations in an expedited way and at a feasible cost for national healthcare systems. In experimental models, certain underlying defects in CFTR mutations have been rectified by administering clinically approved drugs, such as gentamicin (Howard et al., 1996), amlexanox (Gonzalez-Hilarion et al., 2012), escin (Mutyam et al., 2016), ibuprofen (Carlile et al., 2015), and genistein (Illek and Fischer, 1998). These findings indicate that other existing and approved drugs for unrelated disease indications might have the potential to correct or circumvent CFTR dysfunction and should be exploited in the pre-clinical setting. Both cysteamine and thymosin α-1 were also claimed to restore functional expression of F508del-CFTR (Tosco et al., 2016; Romani et al., 2017). Nevertheless, several independent CF research groups failed to demonstrate rescue of F508del-CFTR PM expression and function by either cysteamine or thymosin α-1 (Tomati et al., 2018b; Armirotti et al., 2019; Awatade et al., 2019). Although the immunomodulatory effect of these molecules in CF remains to be further exploited, they did not demonstrate F508del-CFTR correction. As the identification of highly efficient treatments often draws the attention of both scientific and lay audiences, a note of caution should be considered before such findings are diffused in the press to avoid creating premature expectations, especially in CF patients and their relatives.

## Outlook and Conclusion

**Figure 8:**
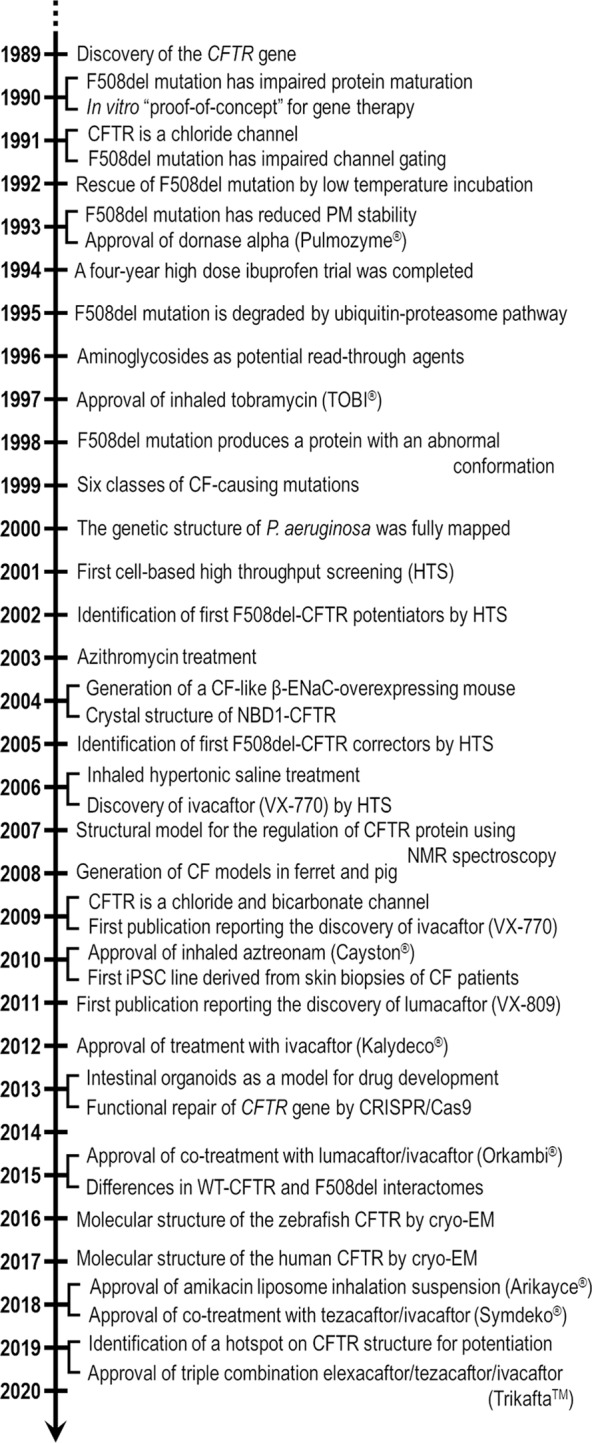
Timeline with several milestones in experimental and clinical research for cystic fibrosis since the discovery of CF transmembrane conductance regulator (*CFTR*) gene in 1989. The knowledge accumulated over these 30 years has been ensuring a better understanding of the molecular biology and protein structure of CFTR, and the pathophysiology of CF in order to translate the basic sciences into clinical practice. More than 10 novel CF therapies have been approved by the U.S. Food and Drug Administration (FDA) during this period with four of these being CFTR modulator drugs.

Thirty years have passed since the discovery of the *CFTR* gene, and numerous milestones in experimental and clinical research of CF have been achieved during this period (**Figure 8**). Understanding the cellular and molecular basis of the disease has paved the way for the development of therapeutic strategies targeting the underlying dysfunctions caused by CF mutations. CFTR modulator therapies are in clinics and they represent a landmark in patients' lives, demonstrating short- and long-term benefits in clinical outcomes. Nevertheless, strategies to achieve optimal, lifelong adherence to treatments should be optimized. Several barriers have still been preventing equitable access worldwide of the current CFTR modulators, including the costs and regulatory national issues, and as such further discussions are needed to identify feasible and sustainable solutions for these therapies to achieve all eligible patients. Furthermore, many rare and ultra-rare CF-causing mutations are still without any efficient, corrective therapy. Novel tools have been developed to accelerate and continue to expand the pipeline of CFTR modulators. From a translational perspective, the use of patient-derived specimens would ensure a comparative evaluation of drug efficacies *in vitro* to select the best candidate(s) and predict the therapeutic responses at an individual level. Every patient is unique, but everyone certainly wants the same: to have a longer and healthier life (ideally, with no symptoms or complications). Hopefully, precision medicine will enable “the highest attainable standard of health” for all patients with CF, and then we will see “all our brothers and sisters breathing free.”

## SOURCE OF DATA

CF patients under care at accredited care centers in Argentina, Australia, Brazil, Canada, Europe, New Zealand, South Africa, United Kingdom, and United States of America. **Figures 2** and **3** are data compiled from the last Patient Registry Report in Australia (Cystic Fibrosis Australia), Brazil (Brazilian Cystic Fibrosis Study Group), Canada (Cystic Fibrosis Canada), Europe (European Cystic Fibrosis Society), New Zealand (Cystic Fibrosis New Zealand), UK (Cystic Fibrosis Trust), and USA (Cystic Fibrosis Foundation). Data from Argentina (Associación Argentina de Lucha contra la Enfermedad Fibroquística del Páncreas) and South Africa (South Africa Cystic Fibrosis Association) have been obtained from Annals of scientific events. A citation with the link to access each Registry Report has been provided.

## Author Contributions

The author confirms being the sole contributor of this work and has approved it for publication.

## Funding

The author is a recipient of the 2018 Gilead Sciences Research Scholars for Cystic Fibrosis.

## Conflict of Interest

The author declare that the research was conducted in the absence of any commercial or financial relationships that could be construed as a potential conflict of interest.
